# Low-carbon diets can reduce global ecological and health costs

**DOI:** 10.1038/s43016-023-00749-2

**Published:** 2023-05-15

**Authors:** Elysia Lucas, Miao Guo, Gonzalo Guillén-Gosálbez

**Affiliations:** 1 Science and Solutions for a Changing Planet DTP, and the Department of Chemical Engineering, Imperial College London, London, UK; 2 Institute for Chemical and Bioengineering, Department of Chemistry and Applied Biosciences, ETH Zürich, Zürich, Switzerland; 3 Department of Engineering, King’s College London, London, UK

**Keywords:** Environmental impact, Climate-change mitigation, Environmental economics

## Abstract

Potential external cost savings associated with the reduction of animal-sourced foods remain poorly understood. Here we combine life cycle assessment principles and monetarization factors to estimate the monetary worth of damage to human health and ecosystems caused by the environmental impacts of food production. We find that, globally, approximately US$2 of production-related external costs were embedded in every dollar of food expenditure in 2018—corresponding to US$14.0 trillion of externalities. A dietary shift away from animal-sourced foods could greatly reduce these ‘hidden’ costs, saving up to US$7.3 trillion worth of production-related health burden and ecosystem degradation while curbing carbon emissions. By comparing the health effects of dietary change from the consumption versus the production of food, we also show that omitting the latter means underestimating the benefits of more plant-based diets. Our analysis reveals the substantial potential of dietary change, particularly in high and upper-middle-income countries, to deliver socio-economic benefits while mitigating climate change.

## Abstract (web summary)

The hidden costs of current diets, or the cost saving associated with the adoption of low-carbon diets, remain unknown. This study combines life cycle assessment and monetarization factors to quantify the indirect costs of nine global dietary change strategies which progressively reduce animal-sourced foods, including consumption-linked health burden from changes in diet-related disease risk.

## Main

Meeting the Paris Agreement targets^1^ will require sharply curbing emissions from a broad range of industrial sectors. The global food system is likely to be strongly affected by this trend as it currently accounts for a third of global anthropogenic greenhouse gas (GHG) emissions^2,3^. Cutting emissions from the food sector involves strategies such as adjusting diets, reducing food waste, improving agricultural practices and increasing resource efficiencies. Notably, dietary change has considerable mitigation potential and would not necessarily require new technology or innovation. For example, reducing the intake of animal-sourced foods (ASF) could yield important climate benefits^3–10^, while contributing to planetary stability regarding land use, biogeochemical cycles, biodiversity and water use^4,11^, as well as improving public health outcomes^6,7,12^. Relative to plant-based foods (PBF), ASF production emits appreciably more carbon per gram^3^ and accounts for the majority of total food production emissions^9^.

Compared with current understanding of the direct climate and environmental implications of dietary changes, their indirect cost repercussions remain relatively under-explored. For instance, the environmental impacts generated by food production incur wider ecological and socio-economic costs, known as externalities, which are not fully reflected in prices paid by producers or consumers^13,14^. Quantifying these externalities via bottom-up^15–19^ or top-down^20–23^ strategies is an important step towards fully understanding the broader implications of dietary change and could provide further scientific evidence for more effective policymaking. However, previous studies on the environmental externalities of diets have limited scope—focusing on single countries^16,17,20–26^, specific food types (fruits and vegetables^26^, ASF^15^ or pork only^17,27^) or individual impact categories (GHG emissions^15,16,18,19^ or nitrogen flows^24^). A more holistic assessment of the worldwide ecological and socio-economic collateral cost implications of changes in food consumption and production levels for decarbonization is therefore still lacking.

**Fig. 1:**
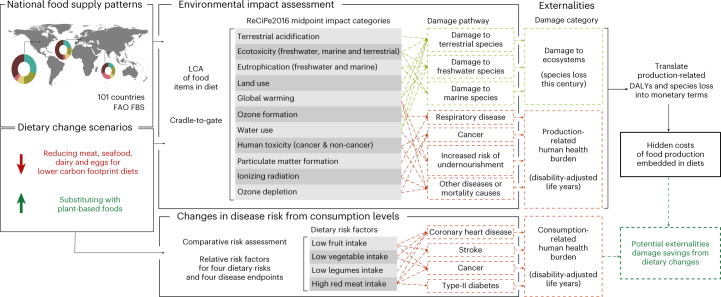
Framework to evaluate the cost of externalities from food production and investigate potential savings from potential hypothetical dietary change scenarios. The global damage to human health (in disability-adjusted life years, DALYs) and ecosystem quality (species loss over the next century) caused by the environmental impacts of food production were examined using a ‘bottom-up’ approach, combining national food supply data and LCIA of individual food items. Each environmental impact category (for example, particulate matter formation, ozone formation) is linked to one or more externality category (health burden and/or ecosystem quality decline) via cause-and-effect damage pathways modelled in the ReCiPe2016 LCIA method^61^ (further details provided in Section 1 of the Supplementary Information). All environmental impacts contributing to each externality category were converted to the respective unit of damage (DALYs or species loss) and then aggregated to form a cumulative damage amount. Total costs of externalities were then estimated by translating DALYs and species loss into their monetary worth. In parallel, changes in disease risk (for four disease endpoints linked to four dietary intake risk factors) were estimated for each dietary change scenario using a comparative risk assessment approach to quantify potential changes in disease-specific DALYs attributable to considered risk factors. World map reproduced using ref. ^6^.

This study fills this gap by quantifying the indirect costs of nine global low-carbon dietary change strategies which progressively reduce ASF groups (Fig. 1). Our holistic analysis of the global externalities of diets combines life cycle impact assessment (LCIA) principles, Food and Agriculture Organization (FAO) Statistics Division Food Balance Sheets (FBS)^28^ and monetarization factors^29,30^ to assess the external costs of the food supplies of 101 countries considering the damage linked to environmental change caused by food production to human health and ecosystems. We find that these external costs of food production embedded in diets worldwide are substantial; by considering nine low-carbon dietary change scenarios, we estimate that these production-linked external costs could be drastically reduced by lowering the proportion of ASF in diets. Our global-level results shed light on the current magnitude and sources of the hidden external costs of food production on the health of the population and ecosystems worldwide—which could be widely reduced by dietary shifts in developed countries. For each scenario, we also estimate the potential reduction in consumption-linked health burden from changes in diet-related disease risk. By considering changes in health burden due to the consequences of both production and consumption, we offer a more holistic appraisal of the effects of dietary change on human health and draw attention to the strong indirect links between diets and human health through the environmental changes caused by food production.

With the approach taken in this study, we aim to progress the science, industry and policy communities towards greater recognition and understanding of the external costs of food. This approach, however, is subject to certain limitations and assumptions which are detailed in Section 10 of the Supplementary Information.

## Results

### Hidden costs of food production embedded in diets

**Fig. 2:**
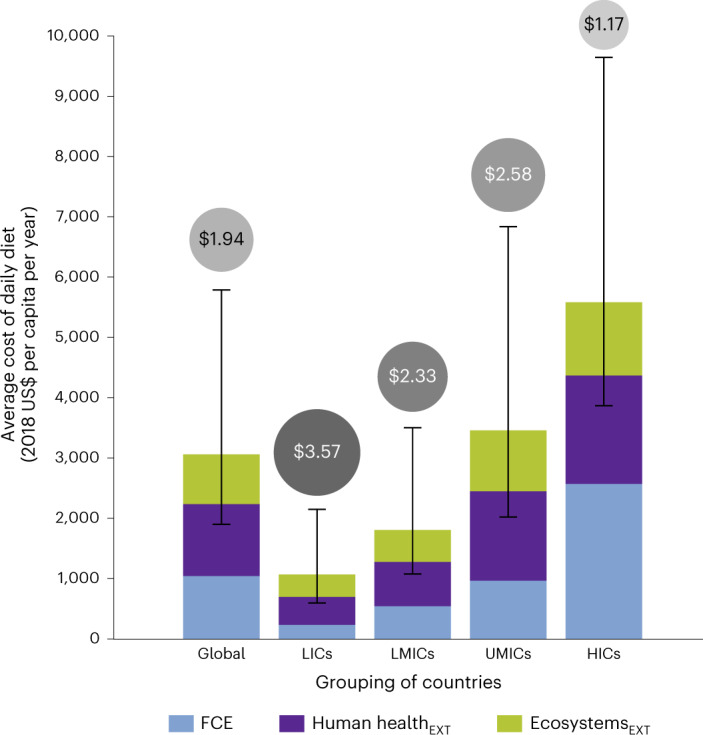
External cost of the production of consumed food and non-alcoholic drink on human health and ecosystems compared with reported final consumption expenditure. Bars present the mean values for consumer cost (final consumption expenditure, FCE) and monetarized human health (Human health_EXT_) and ecosystems (Ecosystems_EXT_) externalities of diets (annual per capita) for all countries in the analysis (global), LICs, LMICs, UMICs and HICs. Error bars indicate lower and upper bounds of the uncertainty range on the average external costs of diets, based on the 95% confidence interval values of life cycle impacts of food items (*n* = 1,000 Monte Carlo simulation runs) and the lower and upper bounds of the monetarization factors of DALYs and species loss. Error bars are not provided on bars showing average FCE, as confidence intervals on expenditure values were not available. Values in bubbles are the mean cost of externalities per US$1 of consumer expenditure on food and non-alcoholic drink consumption (total cost of externalities divided by FCE) for the global, LIC, LMIC, UMIC and HIC groups.

As a first step in gaining deeper insight into the global implications of dietary change, we quantify the external costs of food production in 2018, taken as a baseline. We find that they are worth US$14.0 trillion (US$5.9–32.8 trillion), which is equivalent to 17% (7–39%) of the world’s gross domestic product (GDP) and greater than that of China (Fig. 2; all monetary values expressed in 2018 US dollars). Of the total investigated damage caused by food production, approximately US$8.3 trillion (US$4.9–13.4 trillion) is linked to human health burden and US$5.7 trillion (US$1.1–19.4 trillion) to ecosystem quality decline (Fig. 2). These damage costs are considered to be external, or hidden, costs as the cost implications of disease or premature mortality burden on the wider population and ecological species loss linked to the production of a food item are not accounted for in its consumer price.

Overall, the hidden costs from food production for every dollar paid by consumers in 2018 globally averaged at US$1.94 (US$0.82–4.56) (Fig. 2), corresponding to US$1.15 of production-linked human health burden and US$0.79 of damage to ecosystems. These hidden costs make the ‘true’ total cost of the average global diet nearly triple the amount it costs consumers, or, conversely, consumers are paying on average less than a third of the cost of food if the consequences of food production on health and the environment were accounted for.

**Extended Data Fig. 1:**
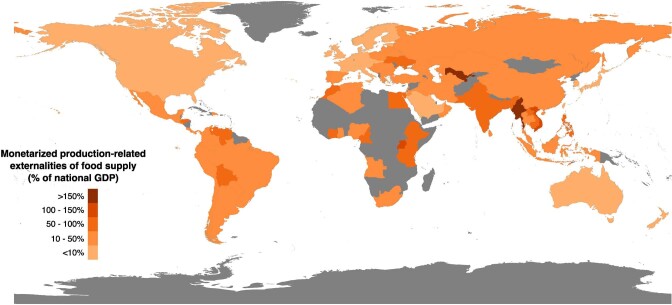
World choropleth map66 showing the estimated monetarized damage to human health and ecosystem quality of national food supplies, expressed as a percentage of national GDP in 2018. Countries shaded in grey are countries not included in analysis.

Consumer and external costs of diets generally increase from low- to high-income group countries, but the ratio of hidden external cost to consumer expenditure shows the reverse trend (Fig. 2). Diets in higher-income regions are found to be the most expensive—in both externalities and consumer cost—due to diet composition and greater average food intake, as well as food product prices. Relative to GDP, however, the economic burden of external costs of food for higher-income countries is less pronounced than for lower-income countries; that is, external cost estimates as a percentage of total GDP range from 7% (3–16%) for high-income countries (HICs) to 102% (43–228%) for low-income countries (LICs) (Extended Data Fig. 1 and Supplementary Table 1).

**Fig. 3:**
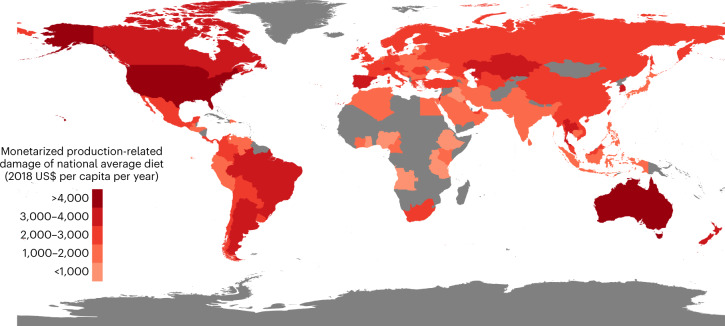
World choropleth map^66^ showing the estimated annual monetarized externalities associated with the average per capita diet of each analysed country (101 countries). Red shades represent the magnitude of the combined externalities on health and ecosystems, linked to the environmental impacts from the production of the national average per capita food supply. By calculating externalities based on per capita food supply, we also include the impacts associated with portions of discarded and wasted food. Countries that are not analysed are shaded in grey.

**Extended Data Fig. 2:**
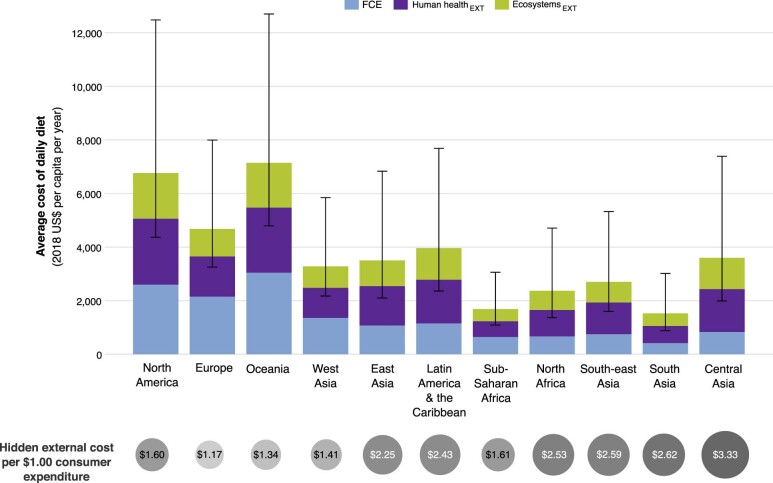
External cost of the production of consumed food and non-alcoholic drink on human health and ecosystems compared to reported Final Consumption Expenditure (FCE) for each geographical region. Bars show the mean consumer cost (FCE) and monetarized human health (Human health_EXT_) and ecosystems (Ecosystems_EXT_) externalities of diet (annual per capita) for each geographical region. Regional grouping of countries can be found in Supplementary Table 12 in Supplementary Data. Cost of externalities per US$1 of consumer expenditure on food and non-alcoholic drink consumption (total cost of externalities divided by FCE) shown in bubbles for each region. Error bars indicate lower and upper bounds of the uncertainty range on the average external costs of regional diets, based on the 95% confidence interval values of life cycle impacts of food items (n = 1,000 Monte Carlo simulation runs) and the lower and upper bounds of the monetarization factors of DALYs and species loss. Error bars are not provided on bars showing average FCE as confidence intervals on expenditure values were not available.

**Extended Data Fig. 3:**
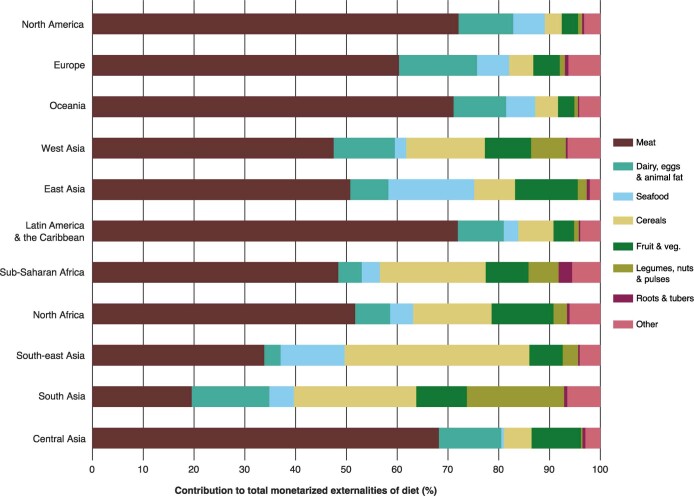
Food group contributions, expressed as percentage of total monetarized externalities associated with the average per capita diet of each geographical region. Bars reflect total combined external costs on health and ecosystems. Total externalities for the average per capita diet of each income group (that is, total of all food group contributions in absolute monetary terms) are presented in Extended Data Fig. 2. ‘Other’ includes contributions from oil crops, oils, stimulants, spices and sweeteners.

In terms of regional distribution, the average diets in North America and Oceania have the greatest annual monetarized externalities per capita (~US$4,200), while the lowest externalities arise from average diets in South Asia and sub-Saharan Africa (~US$1,100) (Fig. 3). Large disparities in annual per capita externalities are found across nations, for example, fivefold increase from Ethiopia to the USA, at US$740 and US$4,380, respectively. For the remaining regions, we uncover more moderate levels of annual external costs associated with their average diets—ranging between US$2,500 and US$2,800 for Europe, Central Asia, East Asia, Latin America and the Caribbean and between US$1,700 and US$2,000 for Southeast Asia, West Asia and North Africa (regional results available in Extended Data Figs. 2 and 3).

Although the average amount of food intake in high-income regions such as North America, Oceania and Europe is greater than in LICs and lower-middle income countries (LMICs), the large gap in externalities we find between higher- and lower-income regions can be mainly attributed to highly differing diet compositions. On a calorie basis, the external costs embedded in the diets of HICs and upper-middle-income countries (UMICs) (US$2.52 and US$2.22 per 1,000 kcal, respectively) are approximately double that of LMICs and LICs (US$1.32 and US$1.02 per 1,000 kcal, respectively). Further details on calorie-level results are provided in Supplementary Tables 2 and 3.

**Fig. 4:**
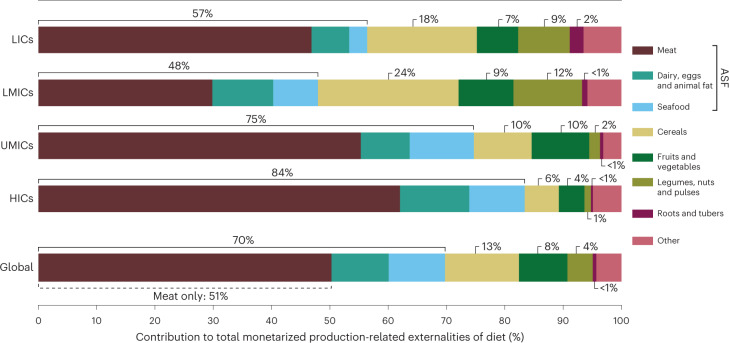
Food group contributions, expressed as a percentage of total monetarized externalities caused by the production of food consumed globally and in LICs, LMICs, UMICs and HICs. Bars represent the total combined external costs on health and ecosystems. Total externalities for the average per capita diet of each income group (that is, total externalities of all food group contributions in absolute monetary terms) are provided in Fig. 2. ‘Other’ includes contributions from oil crops, oils, stimulants, spices and sweeteners.

Breaking down contributions to externalities reveals that the levels of ASF (meat, seafood, dairy, eggs and animal fat) consumption greatly influence the indirect costs of food (Fig. 4). Even though ASF consumption remains relatively modest in lower-income regions, ASF are responsible for a notable share of the total externalities globally—ranging from 48% for LMICs to 84% for HICs, with meat production (beef, pork, lamb and chicken) accounting for 51% of the worldwide total (Fig. 4). Cereals represent another major contributor to the externalities of all income groups, but particularly for lower income where diets are highly dependent on cereal staple crops. By the same token, legumes, nuts and pulses contribute markedly more to the externalities of lower-income countries compared with HICs and UMICs.

### Mitigating externalities of food production through low-carbon diets

We next explore the implications of shifting eating patterns to diets with less ASF. Previous studies^1,4–6,18,19^ concluded that these changes could help mitigate climate change, but they neglected to quantify potential production-related cost implications of wider environmental impacts on important societal aspects such as human health and ecosystems quality. Relative to the 2018 baseline food supply pattern (BASE), we model nine hypothetical dietary change scenarios (Table 1) and apply them to all countries in our analysis. Except for the reference diet proposed by the EAT-Lancet Commission for planetary and human health^4^ (EAT), our scenarios model the implications of progressively lowering amounts of ASF groups from food patterns. They are not, however, intended to reflect exact dietary recommendations. We model the removal of ASF groups from the BASE scenario in progressive stages that reflect how people typically approach transitions to a more plant-based diet^7,31^: no red meat (NRM), pescatarian (PESC), vegetarian (VEG) and vegan (VGN). Calories lost from meat, seafood, eggs and dairy elimination are replaced with legumes, fruits and vegetables. In addition, we consider ‘unconventional’ foods^32,33^, given their recent emergence in Western countries in particular^33,34^. To this end, we model variations of the NRM, PESC, VEG and VGN scenarios in which ASF are partly replaced with insects (for example, mealworms) (NRM with insect substitutions (NRM-I) and PESC with insect substitutions (PESC-I)) or processed plant-based meat (VEG with processed plant-based meat substitutes (VEG-P)) and milk (VGN with processed plant-based meat and milk substitutes (VGN-P)) substitutes (for example, ‘meat’ alternatives derived from mycoprotein, soy, pea or wheat protein and ‘milk’ produced from rice, oat, wheat or nuts).

**Table 1:** Overview of modelled dietary change scenarios

Dietary change scenario	Scenario label	Details	Substitution rule (on calorie basis)
Baseline (2018)	BASE	Based on FAO FBS	−
EAT-Lancet reference	EAT	Recommendations for planetary and human health diet made by the EAT-Lancet Commission^4^	−
No red meat	NRM	Remove beef, lamb and pork	2/3 whole food plant protein (beans, legumes and soybeans), 1/3 fruits and vegetables
No red meat (with insect substitutions)	NRM-I		1/3 whole food plant protein, 1/3 fruits and vegetables, 1/3 insects
Pescatarian	PESC	Remove beef, lamb, pork and chicken	2/3 whole food plant protein, 1/3 fruits and vegetables
Pescatarian (with insect substitutions)	PESC-I		1/3 whole food plant protein, 1/3 fruits and vegetables, 1/3 insects
Vegetarian (with whole foods only)	VEG	Remove meat and seafood	2/3 whole food plant-based protein, 1/3 fruits and vegetables
Vegetarian (with processed food substitutions)	VEG-P		1/3 whole food plant protein, 1/3 fruits and vegetables, 1/3 processed plant-based meat substitutes
Vegan (with whole foods only)	VGN	Remove meat, seafood, dairy and eggs	2/3 whole food plant protein, 1/3 fruits and vegetables
Vegan (with processed food substitutions)	VGN-P		1/3 whole food plant protein, 1/3 fruits and vegetables, 1/3 processed plant-based meat and milk substitutes

Substitution rules are applied with respect to 2018 baseline food supply quantities of each country (BASE).

**Fig. 5:**
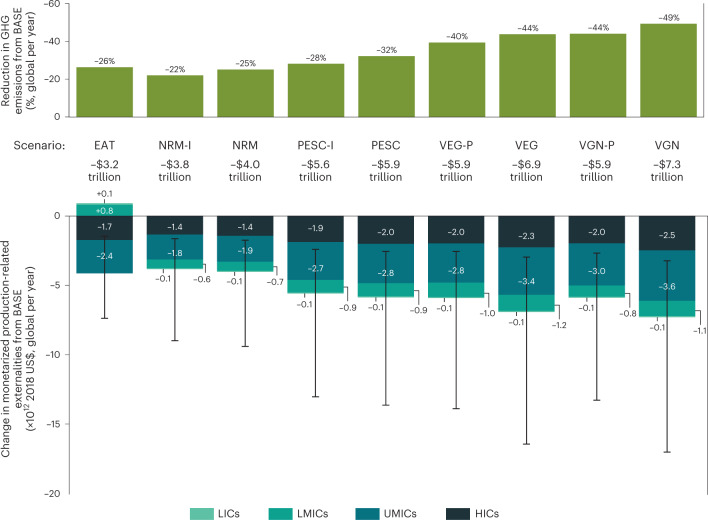
Modelled dietary change scenarios show potential to reduce GHG emissions and deliver savings in production-related externalities. Top: reductions in GHG emissions are expressed as total global percentage reductions from the BASE scenario (2018 food supply patterns, 101 countries). Bottom: change in external costs from BASE scenario are in terms of monetarized damage of food production to health and ecosystems. Bar segments of externalities reduction for dietary change scenarios correspond to the total external cost savings contributions of each income group classification of countries—LICs, LMICs, UMICs and HICs. Error bars indicate lower and upper bounds of the uncertainty range on the total global reduction of external costs based on the 95% confidence interval values of life cycle impacts of food items (*n* = 1,000 Monte Carlo simulation runs) and the lower and upper bounds of the monetarization factors of DALYs and species loss.

We find that substantial savings in both externalities and GHG emissions could be achieved by eliminating ASF from global food patterns to a greater extent (Fig. 5). Removing only red meat (NRM) would reduce externalities and GHG emissions in the range of US$4.0 trillion (US$1.8–9.4 trillion) and 2.3 Gt CO_2_e (2.0–2.8 Gt CO_2_e), respectively. Moreover, the complete exclusion of ASF in the VGN scenario could deliver the largest savings—that is, US$7.3 trillion (US$3.2–17.0 trillion) in externalities and abatement of approximately 4.5 Gt CO_2_e (3.9–5.8 Gt CO_2_e) relative to BASE. The potential savings from the VGN scenario represent 9% (4–20%) of global GDP and 110% (75–280%) of the reduction in GHG emissions from BASE required to meet the food production boundary of the planetary safe operating space for climate change, as derived by the EAT-Lancet Commission^4^.

By far, higher-income regions (UMICs and HICs) hold the most critical role in realizing potential savings in externalities from dietary change. UMICs and HICs account for the vast majority (81–100%) of possible externalities abatement across scenarios (Fig. 5), while eliminating ASF groups in lower-income countries would lead to more modest savings. Consistent with the findings of ref. ^35^, we also find that adopting the EAT diet in LICs and LMICs may risk increasing their GHG emissions which, in turn, could lead to greater externalities.

**Extended Data Fig. 4:**
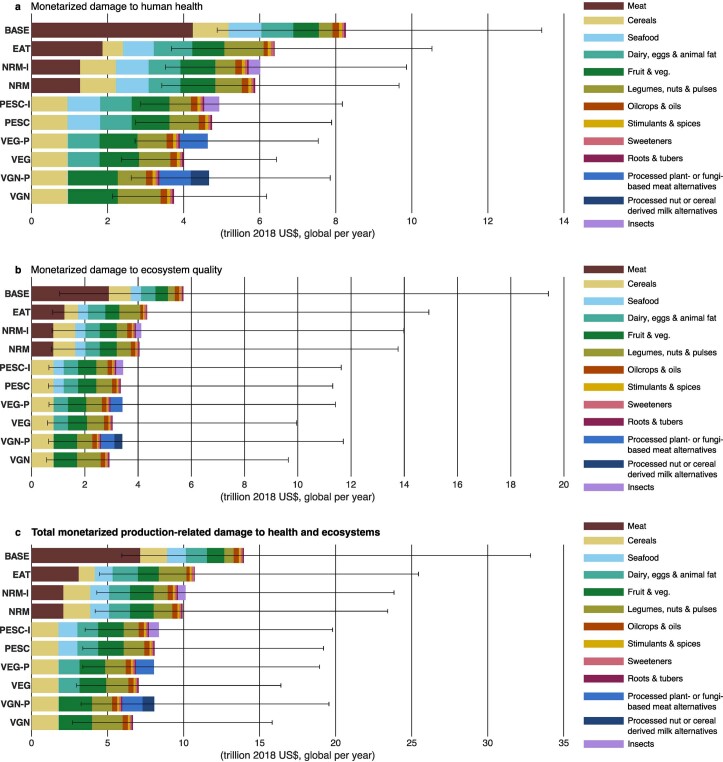
Monetarized damage to human health and ecosystem quality from the environmental impacts of total food supply production (of 101 analyzed countries) for all scenarios. Estimated damage costs are segmented by food group contribution. Panels (**a**) and (**b**) show damage of scenarios to human health and ecosystem quality, respectively. Panel (**c**) presents the combined monetary damage of scenarios to both human health and ecosystems. Constituent food items of each food group are listed in Supplementary Table 9 in the Supplementary Data. Error bars indicate lower and upper bounds of uncertainty ranges on the total global externalities of each scenario based on the 95% confidence interval values of life cycle impacts of food items (n = 1000 Monte Carlo simulation runs) and the lower and upper bounds of the monetarization factors of DALYs and species loss. Scenario *BASE* refers to 2018 food supply; *EAT* is consistent with the EAT-Lancet Planetary Health diet; *NRM-I* and *NRM* refer to food supply with no red meat, substituted by legumes, fruits and vegetables (*NRM*) or legumes, fruits, vegetables and insects (*NRM-I*); *PESC-I* and *PESC* refer to food supply with no meat, substituted by legumes, fruits and vegetables (*PESC)* or legumes, fruits, vegetables and insects (*PESC-I*); *VEG-P* and *VEG* refer to food supply with no meat and animal seafood, substituted by legumes, fruits and vegetables (*VEG*) or legumes, fruits, vegetables and processed meat alternatives (*VEG-P*); and, *VGN-P* and *VGN* refer to food supply with no meat, animal seafood, eggs and dairy, substituted by legumes, fruits and vegetables (*VGN*) or legumes, fruits, vegetables, processed meat and milk alternatives (*VGN-P*).

Our results also show that processed plant-based foods (PPBF) and insect protein could help greatly reduce externalities. Notably, scenarios which partly replace ASF with insect protein (NRM-I, PESC-I) and PPBF (VEG-P and VGN-P) could yield appreciable savings in externalities. However, their associated savings would be smaller than in scenarios substituting ASF with legumes, fruits and vegetables (NRM, PESC, VEG and VGN) (Extended Data Fig. 4). Specifically, the externalities associated with VEG and VGN scenarios are 14% and 21% higher, respectively, when ASF are partly substituted with PPBF instead of legumes, fruits and vegetables. For insects, however, no stark difference was observed in externalities between NRM and NRM-I scenarios.

**Fig. 6:**
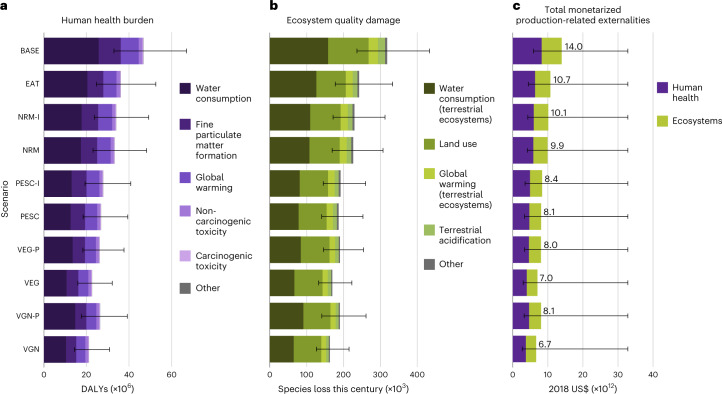
Externalities associated with the production of total food supply in 101 analysed countries for BASE (2018) and modelled dietary change scenarios. **a**,**b**, Further details on the underlying environmental mechanisms or resource use types driving the externalities caused by food production in 2018 and in each dietary scenario. **a**, Estimated damage to human health, expressed in million DALYs, linked to the environmental impacts caused by annual food production for BASE and dietary change scenarios in 101 countries. Stacked bars show the contributions of individual environmental impact category to human health burden. The top five contributing environmental impacts are explicitly shown, and contributions from ‘Other’ include impacts of ozone formation, ionizing radiation and stratospheric ozone depletion. **b**, Estimated damage to ecosystems, expressed in thousands of species loss over the next 100 years, linked to the environmental impacts caused by annual food production for BASE and dietary change scenarios in 101 countries. Stacked bars present the contributions of individual environmental impact category to ecosystem quality decline. The top four contributing environmental impacts are explicitly shown, and contributions from ‘Other’ include impacts of global warming on freshwater ecosystems, ozone formation on terrestrial ecosystems, freshwater eutrophication, marine eutrophication, terrestrial ecotoxicity, freshwater ecotoxicity, marine ecotoxicity and water consumption on aquatic ecosystems. **c**, Total estimated monetarized damage to health and ecosystems, expressed in trillion US$, caused by annual food production for BASE and dietary change scenarios in 101 countries. Error bars indicate lower and upper bounds of uncertainty ranges on total global externalities of each scenario based on the 95% confidence interval values of life cycle impacts of food items (*n* = 1,000 Monte Carlo simulation runs) in **a** and **b**, and on the lower and upper bounds of the monetarization factors of DALYs and species loss in **c**.

We next delve into the topmost environmental impact or resource use types driving externalities damage (Fig. 6a,b). As depicted in Fig. 1, damage to human health and ecosystems caused by food production are modelled in this work as the combined effects of individual environmental impacts (for example, particulate matter formation, ozone depletion).

Lowering the proportion of ASF in supply patterns could avoid a future increase of up to 25.6 million disability-adjusted life years (DALYs) (18.5–36.1 million DALYs) related to environmental change (VGN with respect to BASE) (Fig. 6a). The substitution of meat (PESC), however, accounts for most of this potential (19.9 million DALYs (14.4–27.6 million DALYs)), mainly due to lower agricultural water use demand (Fig. 6a). Meat production requires considerably more water than PBF farming—mostly to grow feed crops^36^. In turn, reducing meat intake could lower water consumption to levels which could prevent increases in undernutrition and food insecurity caused by potential irrigation water shortages^37^.

Similarly for ecosystems, the most appreciable damage savings are achievable from the elimination of meat alone. Elimination of all ASF (BASE to VGN) could prevent 155,000 (110,000–218,000) species loss—most of which is linked to the removal of meat (that is, PESC corresponds to a reduction of 132,000 (95,300–181,000) species loss) (Fig. 6b). Our results indicate that the prevention of species loss through dietary change would mostly be a consequence of lower water consumption and land use (Fig. 6b). Reducing meat intake would avoid drops in freshwater availability which, in turn, would prevent the potential disappearance of terrestrial and freshwater species due to plant diversity decline and river discharge changes^37^. In addition, meat production—especially beef and lamb—is highly land-intensive due to the area required to grow crops for feed as well as for grazing. Curbing meat consumption could free up and restore agricultural land while lessening the demand for new crop (for feed) or pastureland, thus preventing species loss via soil disturbance or habitat loss^37^.

### Diet–health links through food consumption and production

As shown by the results presented so far, dietary shifts could reduce production-linked human health burden by lowering the demand for foods with the highest environmental footprints of production. Food production causes human health burden as there are various pathways in which environmental change can result in negative health outcomes (Fig. 1). The other and most obvious way that dietary change is linked to human health, however, is through consumption of the food itself. For each dietary change scenario, we therefore sought to compare the magnitude of potentially avoided DALYs due to changes in environmental impact levels from food production versus changes in disease risk from adjustments in food intake. The consumption-related health effects of shifting from the BASE diet to each scenario were estimated using a comparative risk assessment approach^18,38–40^ to estimate changes in risk of four disease endpoints (coronary heart disease, stroke, cancer and type-II diabetes) associated with fruit, vegetable, legume and red meat intake (further details are provided in Methods and Section 8 of the Supplementary Information).

Our estimates show that, of the total potential health benefits (in terms of avoided DALYs) from shifting to diets with less ASF and more PBF, approximately a third correspond to the benefits from less environmentally impactful food production. The remaining two-thirds are associated with lower disease risk from consumption (Table 2). Although our findings reinforce the notion that diets are most strongly tied to health via the intake of food itself, they also emphasize that production-linked health effects should be considered when appraising the potential benefits of dietary change. If the health implications of less environmentally damaging food production are neglected, there is a risk of considerably underestimating the health advantages of more plant-based diets. In addition, we note that, unlike direct consumption-related health effects, impacts caused by food production affect the health of others in the wider population—that is, the diet of one person not only affects their own health but also has consequences on the health status of the global and/or national population that are by no means negligible.

**Table 2:** Estimates of reduction in disease and premature mortality burden (expressed in DALYs) from changes in food production and consumption for each dietary change scenario

Dietary change scenario	Potentially avoided DALYs from shifting to diet from BASE (global, millions per year)		
	Total	Production-related	Consumption-related
EAT	30.3 (24.6–33.8)	10.6 (7.5–15.5)	19.7 (17.1–18.3)
NRM-I	37.4 (29.1–44.2)	12.8 (8.7–18.7)	24.7 (20.4–25.5)
NRM	39.2 (30.2–46.7)	13.5 (9.2–19.8)	25.7 (20.9–26.9)
PESC-I	48.3 (36.8–58.6)	18.9 (12.9–27.5)	29.4 (23.9–31.1)
PESC	50.7 (38.3–62.1)	19.9 (13.7–29.0)	30.8 (24.6–33.1)
VEG-P	51.4 (38.3–64.3)	20.6 (13.6–31.2)	30.9 (24.7–33.2)
VEG	57.5 (42.3–73.1)	24.2 (15.9–36.9)	33.4 (26.4–36.2)
VGN-P	67.2 (50.9–81.6)	20.4 (14.3–29.6)	46.8 (36.6–52.1)
VGN	75.5 (55.4–95.0)	25.6 (17.4–38.3)	49.9 (38.0–56.6)

Production-related DALYs refer to the number of DALYs caused by environmental change potentially prevented if food production levels were adjusted to meet the demand of imposed dietary change scenarios. Consumption-related DALYs are estimated DALYs potentially avoided as a result of changes in food intake levels in dietary change scenarios. Uncertainty range values (in brackets) for avoided DALYs related to consumption were estimated using low and high 95% confidence interval values of literature relative risk parameters corresponding to each dietary risk factor–disease pair. Uncertainty range values for avoided DALYs related to production were estimated using the lower and upper 95% confidence interval values of food life cycle impacts (*n* = 1,000 Monte Carlo simulation runs).

## Discussion

Our results uncover the high external costs of food production to ecological and socio-economic systems that are currently embedded in global eating patterns. Measuring the broader damage of food, which is ordinarily overlooked, can more comprehensively illuminate hidden economic hotspots in the negative consequences of food production and consumption while informing decision-making at the individual, business and policy levels. At the same time, monetarizing this wider damage allows overall interpretation of externality damage categories—which are usually measured in different units—while enabling the comparison of the ‘true cost’ of food with its price. Externalities could also be used as sustainability indicators by producers and companies for communication to consumers via, for example, labelling and for price internalization. Although their advantages are clear, the universal implementation of an externalities accounting framework faces many challenges. Reaching consensus on standardized methodologies across organizations will require extensive collaboration^14^. For instance, life cycle assessments (LCAs) are subject to uncertainties and subjective methodological choices^41^, which greatly affect analyses’ outcomes. Furthermore, general acceptance of how to derive the monetary worth of biodiversity loss and human life years will not be straightforward either, given ethical and practical difficulties. Until a harmonized framework for comprehensively assessing externalities is agreed upon, however, the results of our study stand to expand current knowledge of production-related dietary externalities by providing insight into a wider breadth of environmental and socio-economic domains.

Mitigating climate change through dietary change has the potential to also reduce wider externalities caused by food production. Our results indicate that lowering portions of ASF in global dietary patterns would not only reduce GHG emissions but also substantially lessen the damage to health and ecosystems caused by the environmental impacts of agriculture. Our findings therefore reveal that dietary changes motivated by climate change mitigation could also result in accompanying benefits to human health and ecosystem quality. Through translating such damage savings into monetary value, our findings shed light on the economic incentive to facilitate a transition to low-carbon diets. Furthermore, most of the production-caused externalities we find to be embedded in diets correspond to human health damage (Fig. 2), providing evidence that diets and health are strongly linked not only directly through consumption but also indirectly through human exposure to the environmental changes caused by food production.

Although our analysis shows that lowering ASF proportions in diets on a global scale offers large ecological and socio-economic benefits, most of these gains are achievable through changes to the dietary patterns of HICs and UMICs (Fig. 5). Higher-income regions currently consume distinctly more ASF than lower-income regions, and the per capita food production-related externalities in HICs and UMICs are two to three times greater than those of LICs and LMICs (Fig. 2). Ensuring the availability, accessibility, affordability and appeal of less environmentally intensive PBF is, therefore, essential to facilitating shifts to diets with lower production-related external costs. Although our results point to a VGN diet for maximum externalities savings, we note that ASF are likely to remain in the diets of developed countries, albeit in lower proportions, for, for example, food security, livelihood and cultural reasons. Developing producers’ capacity to adopt sustainable livestock practices^42^—which have the potential to improve soil quality and sequester carbon^43^—will, therefore, be key. A mainstream farming transition, however, is unlikely to happen in a short timeframe and from producer initiative or consumer demand alone, therefore warranting government support and legislation. Furthermore, careful policy design will require consideration of the labour market effects that large-scale dietary change could trigger.

Compared with HICs and UMICs, the diets of developing regions do not account for a large portion of global production-related externalities (Fig. 5). Nevertheless, we modelled a global transition away from ASF from a theoretical perspective to compare the impact mitigation potential of dietary changes in LICs and LMICs versus UMICs and HICs. As prevalence of undernourishment and nutritional deficiencies are of major concern in LICs and LMICs^44^, increasing access to healthy and diverse diets is more appropriate than reducing ASF consumption. However, as we found for the EAT diet (Fig. 5), adoption of more diverse and nutritious diets may increase the production-caused externalities of these countries. Limiting increases in externalities would therefore involve greater focus on the implementation of more environmentally sustainable production practices of all foods by ensuring farmers’ access to knowledge, resources and technology. As is also the case for higher-income regions, less environmentally intensive animal farming methods are particularly critical in developing countries as many communities depend on small-scale livestock farming for food and livelihood security, and increases in meat demand are expected to accompany income growth^45–47^.

The incorporation of PPBF (such as imitation meat and milk) and edible insects into diets of HICs and UMICs could help individuals move away from ASF and reduce the externalities from their diets. In Europe, for instance, PPBF have become increasingly popular, and their consumption is expected to continue^34,48^. We note, however, that substituting ASF with PPBF may not reduce externalities as much as replacing them with only unprocessed whole PBF could (Fig. 5). Moreover, given their high level of processing, opting for PPBF over unprocessed whole foods might not be as healthy or nutritious. Nevertheless, substituting ASF with their processed imitation counterparts could facilitate the transition to a more plant-based diet as individuals could continue to eat and cook familiar dishes without major changes to preparation, taste or texture. Furthermore, edible insects offer a promising alternative to red meat and poultry—for example, mealworms are comparable to meat in terms of their protein, vitamin and mineral content^49^. Although insects are already commonly consumed in some regions, they are not yet accepted worldwide. In western countries especially, the ‘disgust’ factor^50^ is a key barrier to overcome. Strategies to normalize insect consumption could include promotion of their environmental and nutritional benefits^32,33,49^ and increased availability of insect-based products that could be readily incorporated into current eating habits with little effort, such as protein bars or insect flour^50^.

The low-carbon diet strategies we model rely on widespread behavioural change in HICs and UMICs that are likely difficult to achieve. Efforts to mitigate production-related food externalities should therefore also encompass strategies that do not depend on changes in consumer attitudes and habits. With focus on the individual environmental impact drivers we find to dominate health and ecosystem externalities of agriculture (Fig. 6a–c), external costs could also be reduced via changes in agricultural practices and operations to reduce water and chemical inputs, land occupation, and direct and indirect GHG emissions, as well as decreased dependence on fossil energy sources. Lowering water, land and chemical inputs to ASF production could be achieved through sustainable intensification^51^—for example, adopting agroecological farming methods including agroforestry, organic farming and integrated pest management. In terms of livestock farming, GHG emissions could be reduced via feed additives to reduce methane from ruminants and the replacement of imported soy for feed with less land-intensive alternatives that are sourced locally. Decoupling food production from fossil resources, also reducing GHG emissions, requires a switch to renewable energy sources and fuel in fishing and on-farm operations, as well as agrochemical manufacturing.

## Conclusions

Our findings support the view that dietary change, as part of a wider food system transformation^52–54^, can bring substantial collateral benefits that are not usually considered in standard assessments. More specifically, dietary shifts towards lower ASF proportions—in line with broad climate change mitigation recommendations—have the potential to considerably alleviate damage caused by food production to human health and ecosystem quality, in turn leading to substantial indirect external cost savings. Our study shows that analysing such ‘hidden’ external costs embedded in current food consumption and expenditure patterns is therefore critical to understanding the broader societal implications of dietary change. On a practical side, the potential of such changes to preserve the integrity of ecosystems and human health, contextualized in terms of monetary worth in this work, should serve as an incentive to spur a widespread dietary transition in developed countries that currently seem to have the most environmentally damaging food consumption patterns.

## Methods

### Food supply and expenditure

We compiled data on national food supply (kg per capita per year and kcal per capita per day) from the FAO FBS^28^ for 2018. Food (including non-alcoholic beverages) supplies for each country are described in terms of 90 food groups in the FBS and reflect the total supply (from domestic production and imports) for that year available for human consumption. We acknowledge that quantities of food supply per capita are not the quantities necessarily consumed, due to, for example, peeling, cooking or waste. However, using food supply data in this analysis allows the entire environmental footprint of food consumption to be captured, and these data are, nevertheless, indicative of the typical diet of each country. Per capita food supply is, therefore, used as a proxy for the average diet. In addition, we assume that the relative proportions of imported and domestically produced food remains constant when scaling national supply down to the average per capita diet^55^.

Data on national final consumption expenditure (FCE) on food and non-alcoholic beverages in 2018 are from the Economic Research Service from the US Department of Agriculture^56^. We note that the FCE data available from the US Department of Agriculture Economic Research Service is only for expenditure on food and non-alcoholic beverage consumed at home. Actual expenditure is, therefore, expected to be higher when factoring in expenditure on food and drink consumed outside of the home. Our estimates of the ‘hidden’ cost factor (that is, cost of externalities per US$1 consumer expenditure) using household only FCE data are likely to be lower than if the factors were to be calculated using expenditure on total (inside and outside of the home) consumption. Furthermore, we only calculate an average hidden cost factor for each country, but, in reality, the hidden cost factor is likely heterogeneous as expenditure varies across socio-demographic groups. Although we could not provide a more detailed breakdown of the hidden cost factor within each country, our results still allow overall cross-national comparisons.

Countries were chosen based on whether they had available food supply data from the FAO FBS, as well as standardized food and drink FCE data, leading to a total of 101 countries (accounting for 91% of the global population in 2018; the full list of countries is in Supplementary Table 11).

### LCIA of food items

We used LCA to calculate the impact intensities (impact per kilogram of food) of 708 food items, with country-specific or global average production methods, using life cycle inventory (LCI) data from the ecoinvent version 3.5 (refs. ^57,58^), Agri-footprint version 4.0 (ref. ^59^) and Energie-Stoffe-Umwelt (ESU) World Food^60^ databases.

For each food item, its ‘midpoint’ (that is, individual environmental impact; for example, global warming potential or water use) and ‘endpoint’ impact (that is, contribution to health and ecosystem burden) intensities were calculated. The externalities considered in this analysis correspond to the damage caused to human health (measured in the number of years lost due to ill-health, disability or death; DALYs) and ecosystem quality (measured by time-integrated species loss; species loss over the next 100 years). Midpoint environmental impacts of food production are their direct effects on the environment via emissions or resource use, and the consequent effect of these midpoint impacts on health or ecosystems (‘endpoints’) can be viewed as further collateral damage.

To calculate both environmental midpoint and externality endpoint impact intensities, we used the SimaPro software (version 9.1.0.8) that implements the ReCiPe2016 LCIA method^37,61^ (with the hierarchic cultural perspective, which models the effects of environmental impact mechanisms over a 100 year time horizon, based on scientific consensus). Using SimaPro, the LCI of each food item (LCI flows generally refer to raw material, energy and water inputs; and direct emissions released to water, soil and air) is converted into a particular midpoint environmental impact using the characterization models of the ReCiPe2016 method. Equation (1) describes the conversion of the inventory flows of a food item’s life cycle into a midpoint environmental impact.

1 $$i_{f,m}^{\mathrm{IMP}} = \mathop {\sum}\limits_j {\mathrm{LCI}_{f,\,j}{\mathrm{CF}}_{j,m}^{\mathrm{IMP}}\forall f \in {\mathrm{FI}},m \in M}$$

where *f* is the index denoting members of set FI of food items available in LCI databases, index *m* denotes members of set *M* of ReCiPe2016 midpoint impact categories, $i_{f,m}^{\mathrm{IMP}}$ is the impact intensity of 1 kg of food *f* for midpoint impact category *m*, LCI_*f*,*j*_ is the LCI input or output of elementary flow *j* from the life cycle of 1 kg of food item *f* and $\mathrm{CF}_{j,m}^{\mathrm{IMP}}$ is the ReCiPe2016 characterization factor used to convert elementary flow *j* to unit impact of midpoint category *m*.

The ReCiPe2016 LCIA method also models cause-and-effect damage pathways linking each midpoint environmental impact to externality damage. Equation (2) outlines how each midpoint environmental impact of a food item can be translated into externality damage using ReCiPe2016 characterization factors (for example, characterization factor to convert global warming potential, in kg CO_2_e, to its estimated human health burden, in DALYs).

2 $$i_{f,e}^{\mathrm{EXT}} = i_{f,m}^{\mathrm{IMP}}{\mathrm{CF}}_{m,e}^{\mathrm{EXT}}\forall f \in {\mathrm{FI}},\,e \in E$$

where index *e* denotes members of set *E* of ReCiPe2016 endpoint, or externality, impact categories, $i_{f,e}^{\mathrm{EXT}}$ is the impact intensity of 1 kg of food *f* for externality/endpoint impact category *e* and ${\mathrm{CF}}_{m,e}^{\mathrm{EXT}}$ is the characterization factor used to convert unit impact of midpoint category *m* to unit impact of endpoint category *e*.

The total externality impact intensity of a food item is the cumulative impact of its related midpoint environmental impacts. The links between each environmental impact and externality types are visualized in Fig. 1, and further details on their specific cause-and-effect damage pathways are provided in Section 1 of the Supplementary Information. To avoid making assumptions regarding retail (for example, transport, energy inputs) and use (such as cooking and preparation) stages, which would introduce a greater level of uncertainty into our calculations, we adopted a cradle-to-gate life cycle approach. As most of the environmental impacts from food are attributed to the farm stage^3^, our approach is expected to have captured the majority of each food item’s footprint. However, excluding the end-use phases from life cycle impact calculations means that our results likely provide a lower bound on total impacts and externalities of global diets in the 2018 baseline and all dietary change scenarios. In addition, our results likely underestimate the difference between the impacts of less versus more developed countries as post-production stages, such as retail and packaging, play a greater role in the food systems of higher-income regions.

The extent of processing varies across food items and, therefore, cradle-to-gate encompasses different activities for each food group. Specifically, cradle-to-gate translates to cradle-to-farm-gate for whole PBF items (for example, fruits, vegetables, cereals, legumes). Cradle-to-slaughterhouse-gate applies to meat; cradle to factory-gate to oils, coffee, tea, cocoa bean, butter, cream, animal fat, fish oil, meat and milk substitutes and cradle-to-harbour to seafood (further details on the stages included in system boundaries are outlined in Supplementary Fig. 1).

### Production-caused impacts and externalities of diets

Each food item taken from the LCI database was matched to its corresponding FAO FBS food group based on its specific crop or animal product type (full list available in Supplementary Table 8). We also calculated the life cycle impact intensities of mealworms, 7 processed meat alternatives and 11 milk alternatives.

Different impact intensities were used for supplied food from domestic production and from imports. To calculate impacts associated with supply from domestic production, country-specific impact intensities were used if they were available. If they were not available, the global average of impact intensities of all available food items corresponding to each FBS food group was used. For imported food impacts, calculations used the global export-weighted average of the impact intensities of all representative food items in each FAO FBS food group.

For many countries, global average intensities were used to calculate the impacts from domestic production due to large gaps in our LCI dataset. It should be noted that these global averages are highly skewed by the relative over-representation and under-representation of production in certain regions. In addition, variation across production methods is not reflected in some food groups with scant data globally. Cereals had the highest representation in our dataset, followed by oil crops and oils; fruits and vegetables; and legumes, nuts and pulses. For all foods, mainly LCI data for production in HICs were available (Europe, in particular). Further details on the food group and geographical coverage of the LCI dataset we used in our analysis is provided in Supplementary Tables 12 and 13.

Hence, for each endpoint impact and externality category and country, the impact of the total supply of each FBS food group is calculated via equation (3) if a country-specific LCI entry is available or equation (4) if no country-specific LCI entry is available:

3 $${\mathrm{Impact}}_{c,g,e} = \frac{{\mathop {\sum}\nolimits_{f \in {\mathrm{FI}}_g} {i_{c,\,f,e}^{\mathrm{EXT}}} }}{{\left| {\mathrm{FI}_{c,g}^{\mathrm{DOM}}} \right|}}{\mathrm{domestic}}_{c,g} + i_{g,e}^ \ast {\mathrm{import}}_{c,g}\,\forall c \in C,g \in {\mathrm{FG}},e \in E$$

4 $${\mathrm{Impact}}_{c,g,e} = \frac{{\mathop {\sum}\nolimits_{f \in {\mathrm{FI}}_g} {i_{c,\,f,e}^{\mathrm{EXT}}} }}{{\left| {\mathrm{FI}_{c,g}^{\mathrm{GLO}}} \right|}}{\mathrm{domestic}}_{c,g} + i_{g,e}^ \ast {\mathrm{import}}_{c,g}\,\forall c \in C,g \in {\mathrm{FG}},e \in E$$

where *c* is the index denoting members of set *C* of countries, *g* is the index denoting members of set FG of FBS food groups, Impact_*c*,*g*,*e*_ is total impact in endpoint category *e* of supplied FBS food group *g* in country *c*, $\left| {\mathrm{FI}_{c,g}^{\mathrm{DOM}}} \right|$ is the number of food items specific to country *c* corresponding to FBS food group *g*, $\left| {\mathrm{FI}_{c,g}^{\mathrm{GLO}}} \right|$ is the number of food items corresponding to FBS food group *g* for all countries, domestic_*c*,*g*_ is the supply of FBS food group *g* in country *c* that is domestically produced, $i_{g,e}^ \ast$ is export-weighted average impact intensity of all food items corresponding to FBS food group *g* for externality or endpoint impact category *e* and import_*c*,*g*_ is the imported food supply of FBS food group *g* in country *c*.

For each impact category and country, the impact of total food supplied is calculated via equation (5):

5 $${\mathrm{Impact}}_{c,e}^{\mathrm{TOT}} = \mathop {\sum}\limits_{g \in {\mathrm{FG}}} {\mathrm{Impact}_{c,g,e}\forall c \in C,e \in E}$$

In our consumption-based approach, impacts and externalities of imported food are allocated to importing countries (equations (3) and (4)) because the main focus of this study was the magnitude of externalities embedded in diets. In the context of this work, we, therefore, adopt the perspective that the diets or food consumption patterns of the importing country are those demanding those traded foods, and those food impacts should be allocated to the diets that have driven their production. We also take a general global view of production-caused externalities from diets, meaning that the externalities caused by diets are embedded in their costs, regardless of where they occur in the world.

### Monetarization of externalities

Monetarization of damage to human health and ecosystems involved the conversion of DALYs and species loss to monetary terms using previously adopted monetarization factors for externalities calculated using ReCiPe2016^29,30,62,63^.

In line with the ‘budget constraint’ method, the monetary value of 1 DALY is equated to the potential average annual economic production of a person at full well-being^29^, where the potential average annual economic production per capita is based on the GDP of the USA—the largest economy in the world. The monetarization factor for human health damage in our analysis is calculated via equation (6) and is valued at US$176,624 per DALY, with a lower bound of the uncertainty range of US$148,288 and upper bound of US$200,236 (all monetary values are in 2018 US dollars).

6 $${\mathrm{MF}}_{\mathrm{HH}}^{2018} = \alpha \left( {\mathrm{GDP}^{2018} + {\mathrm{GHP}}^{2018}} \right)$$

where GDP^2018^ is the 2018 GDP per capita of the USA based on purchasing power parity (PPP), GHP^2018^ is the gross household production in the USA in 2018 (estimated at half of GDP) and *α* is an adjustment factor of 1.87 (uncertainty range of 1.57–2.12). The latter allows the potential average annual economic production per capita to account for impacts on economic production from unemployment, underemployment, health issues, trade barriers and insufficient education^29^.

Damage to ecosystem quality, in the context of this analysis, was valued on the basis of its equivalent reduction in human well-being or, in other words, ‘what sacrifice in terms of disabilities or lost life years would be acceptable’ for ecosystem protection, based on choice modelling^29^. As the monetarization factor for ecosystem damage derived by ref. ^29^ was initially applied to an analysis for the year 2000 (ref. ^30^), we applied the benefit transfer concept^64^ to account for the effect of income growth from 2000 to 2018 on the valuation of ecosystem protection:

7 $${\mathrm{MF}}_{\mathrm{ECO}}^{2018} = {\mathrm{MF}}_{\mathrm{ECO}}^{2000}\left( {\frac{{\mathrm{GDP}^{2018}}}{{\mathrm{GDP}^{2000}}}} \right)^{{\it{\epsilon }}_{\mathrm{ECO}}}$$

where $${\mathrm{MF}}_{\mathrm{ECO}}^{2000}$$ is the monetarization factor for the ecosystems damage endpoint impact ECO applied for the year 2000, GDP^2000^ is the US GDP per capita based on PPP in 2000, GDP^2018^ is the US GDP per capita based on PPP in 2018 and *ϵ*_ECO_ is the income elasticity of willingness to pay for ecosystem protection. The income elasticity of willingness to pay for ecosystem protection reflects the change in how much society is willing to pay for the protection of 1 species with a change in income and is estimated at 0.38 (ref. ^65^). After accounting for income growth, the monetarization factor for time-integrated species loss in our calculations was US$17,891,594 per species loss, with a lower bound of US$4,472,899 and an upper bound of US$44,728,985.

The monetarization of the endpoint impacts of each country’s food supply was, therefore, calculated via:

8 $${\mathrm{Cost}}_{c,e}^{\mathrm{EXT}} = {\mathrm{Impact}}_{c,e}^{\mathrm{TOT}}{\mathrm{MF}}_e^{2018}\,\forall c \in C,e \in \left\{ {{\mathrm{HH}},{\mathrm{ECO}}} \right\}$$

### Dietary change scenarios

Nine hypothetical dietary change scenarios were modelled by adjusting the 2018 national supply quantities of FBS food groups to reflect each diet: the healthy EAT (ref. ^4^), NRM, NRM-I, PESC, PESC-I, VEG, VEG-P, VGN and VGN-P.

The EAT diet is specified in terms of the recommended daily intake for 21 food groups—aiming to provide a total of 2,503 kcal per day (corresponding to the average energy requirements of a 30-year-old woman weighing 60 kg, with moderate to high physical activity level)^4^. To model shifts to the EAT diet in each country, we aggregated the 21 food groups into 15 categories (greater detail provided in Section 2 of the Supplementary Information) for better harmonization with FBS food groups. We then converted EAT diet intake amounts into supply quantities using edible portion factors for different food types^38^ and scaled the per capita supply quantities of each country to the supply quantities of the EAT diet for each food category, maintaining the portion of constituent FBS food groups.

In the remaining scenarios, dietary changes were modelled by eliminating ASF groups from the supply of each country and replacing the lost calories from ASF with legumes, fruits, vegetables, insects and processed plant-based meat and milk alternatives, in varying proportions—as described in Table 1. The total number of calories supplied per capita for each country was kept constant across scenarios (excluding the EAT diet) to isolate the effects of ASF group removal. Replacing calorie contributions from ASF groups with two-thirds beans, legumes and soybeans and one-third fruits and vegetables for the NRM, PESC, VEG and VGN scenarios was based on a previously adopted ASF dietary substitution rule^7^. We maintained the proportion of constituent FBS groups in 2018 supply when scaling up the supply quantities of beans, legumes, soybeans, fruits and vegetables to compensate the lost kilocalorie from ASF.

For the NRM-I, PESC-I, VEG-P and VGN-P scenarios, we assume that individuals would view protein from insects and processed meat and milk alternatives to be analogous to whole food plant-based proteins (beans, legumes and soybeans) in terms of dietary and taste function.

Due to LCI data availability, we could only use mealworm (larvae of the mealworm beetle) farming to estimate the environmental impact intensity of edible insect production. Other common insects for human consumption include crickets, housefly larvae and black soldier fly larvae^32^. Strictly speaking, edible insects are ASF; yet, in the context of this analysis, they are considered separately as human consumption of insects is not yet globally widespread^50^ and insect farming methods are remarkably different from traditional livestock farming^33^. Insect food products are also generally viewed as alternatives to red meat and poultry and do not appeal to vegetarians or vegans^50^. Hence, we only incorporate insects for dietary scenarios where they substitute red meat and/or poultry.

To estimate the impact contributions from the substitution of ASF with processed plant-based meat and milk alternatives, we used average impact intensities for all processed meat alternatives (protein derived from fungi (mycoprotein), soybean, pea, egg, wheat and whey) and for all milk alternatives (almond, cashew, oat, rice, soy and spelt based). In this study, we consider the term ‘plant-based’ to encompass foods derived from plants or fungi.

Using the adjusted national supply quantities of food groups, the midpoint environmental and endpoint externality impacts were then re-calculated for each country to analyse changes across dietary change scenarios. When modelling the effects of dietary change on the environmental impacts caused by production, we assumed that agricultural practices and cultivation patterns would remain unchanged—that is, the impact intensities of food would not change with changes in total production quantities. We, therefore, did not account for any potential feedbacks in the production system which may arise from dietary change. Although such complex system dynamics are not within the scope of this work, increased demand for certain crops, for instance, may result in the expansion of its production to less suitable land areas which could require more resource inputs such as water and fertilizers, thus increasing their impact intensities. Conversely, increased demand for, for example, insect products could allow insect farming to achieve economies of scale and require less energy and resource inputs per kilogram of production.

### Health effects of changes in dietary intake

We estimated the effects of changes in food consumption on human health using an established comparative risk assessment approach^18,38–40^. We considered four major dietary risk factors related to food intake (low intake of fruits, vegetables and legumes and high intake of red meat) and their effect on the risk of developing coronary heart disease, stroke, cancer and type-II diabetes. For each dietary risk factor–disease pair, we estimated the population-attributable fraction (PAF)—representing the proportion of disease cases that would be reduced if risk factor exposure (in this case, food intake) were reduced to an alternative ideal exposure scenario—via the following general formula:

9 $${\mathrm{PAF}}_{i,d} = \frac{{{\int} {\mathrm{RR}_{i,d}\left( x \right)P_i\left( x \right)dx - {\mathrm{RR}}_{i,d}\left( {\mathrm{TMREL}} \right)} }}{{{\int} {\mathrm{RR}_{i,d}\left( x \right)P_i\left( x \right)dx} }}$$

where RR_*i*,*d*_(*x*) is the relative risk of disease (*d*) for dietary risk factor (*i*) at level *x* as modelled in each dietary scenario, *P*_*i*_(*x*) is the proportion of the population exposed to dietary risk factor (*i*) at level *x* in each dietary scenario and TMREL is the theoretical minimum risk exposure level assumed for the alternative ideal exposure scenario corresponding to the level of intake associated with the lowest risk of disease burden. Further details on the relative risk parameters for each dietary risk–disease pair and their sources can be found in Section 8 of the Supplementary Information. For all dietary scenarios in our study, we assume that the entire population in each country is subject to the risk factor level *x* (average intake of risk factor food group) in the scenario of interest.

For diseases with multiple attributable dietary risks, we assumed that risk factors are not correlated and their overall effect on a disease can be evaluated via:

10 $${\mathrm{PAF}} = 1 - \mathop {\prod}\limits_i {\left( {1 - {\mathrm{PAF}}_i} \right)}$$

We then estimated the number of DALYs attributable to the dietary risks considered by multiplying the PAF for each disease by the total number of disease-specific DALYs in 2018 in each country. The number of DALYs we estimate to be attributable with changes in intake-related dietary risks were not monetarized in our study as the emphasis of our study is on the embedded cost in food prices—that is, costs incurred up to point of purchase that is ‘hidden’ in the price. In other words, the cause of a change in human health effects from the environmental impacts of food production has already occurred when a consumer purchases their food, while the cause of a change in health effects from consumption occurs after the purchase.

### Scenario data

The set of dietary scenario results (including the 2018 BASE) included estimates of 22 ReCiPe2016 midpoint environmental and 3 ReCiPe2016 endpoint externality impacts^61^ of the food supplies of 101 countries. Midpoint environmental impact values were analysed in units of their respective externality damage type, while externalities were compiled in both absolute (DALYs and time-integrated species loss, for human health and ecosystem quality, respectively) and monetary units. Global warming potential (a ReCiPe2016 midpoint environmental impact) of scenarios were also analysed in units of kg CO_2_e to study GHG emission changes. Each dietary change scenario (excluding the 2018 BASE) also had the number of potentially avoided DALYs from reduction in disease risk due to changes in food intake with respect to the 2018 BASE.

### Uncertainty

Lower and upper bound estimates quoted for production-related externalities calculated via LCA are based on uncertainty ranges of impact intensity data, as well as uncertainty ranges of monetarization factors for human health and ecosystem damage^29^. To account for the uncertainty associated with LCI data, Monte Carlo analysis (an in-built function in SimaPro 9.1.0.8 software) was performed to obtain lower and upper 95% confidence interval values for the impact intensity of each food item. Each Monte Carlo simulation ran 1,000 iterations, whereby values were randomly sampled for each LCI data input based on its specified uncertainty distribution parameters available in the software. Lower and upper estimates of attributable DALYs for each dietary risk factor–disease pair in the comparative risk assessment are based on the lower and upper 95% confidence interval values of relative risk parameters (values are given in Section 8 of the Supplementary Information).

### Reporting summary

Further information on research design is available in the Nature Portfolio Reporting Summary linked to this article.

## Supplementary information

Supplementary Information Supplementary Figs. 1–11, Supplementary Tables 1–7 containing additional results and parameters used in comparative risk assessment.

Reporting Summary

Supplementary Data 1 Supplementary Tables 8–13 containing select input data and description of geographical and food item coverage of dataset.

## Data Availability

Food supply quantities and externalities results for all dietary change scenarios modelled in this study are available at https://github.com/eglucas/LowCarbonDiets_Externalities. Select input data are also available in Supplementary Tables 8–13. Food supply data used in this study are available from the FAO Food Balance Sheets (http://www.fao.org/faostat/en/#data/FBS), and FCE data are available from the US Department of Agriculture Economic Research Service (https://www.ers.usda.gov/topics/international-markets-us-trade/international-consumer-and-food-industry-trends/#data). Life cycle inventory data for the calculation of food item impacts can be accessed in the commercially available databases ecoinvent (https://ecoinvent.org/the-ecoinvent-database/), Agri-Footprint (https://blonksustainability.nl/tools/agri-footprint) and ESU World Food (https://esu-services.ch/data/fooddata/).

## Code availability

Code to calculate environmental impacts and externalities of scenarios is available at https://github.com/eglucas/LowCarbonDiets_Externalities.
