# Supplementary material for: Low-carbon diets can reduce global ecological and health costs
Source: Nat Food. 2023 May 15;4(5):394–406. doi: 10.1038/s43016-023-00749-2 (PMC10208974; doi:10.1038/s43016-023-00749-2)
Supplement: Supplementary file 1 — Supplementary Figs. 1–11, Supplementary Tables 1–7 containing additional results and parameters used in comparative risk assessment. [file 43016_2023_749_MOESM1_ESM.pdf]

nature food

Article https://doi.org/10.1038/s43016-023-00749-2

Low-carbon diets can reduce global ecological and health costs

In the format provided by the authors and unedited

This document includes supplementary material, methods and results.

- Section 1 provides further detail on the activities included in the cradle-to-gate life cycle of food items, as well as a simplified overview of the damage pathways modelled in the ReCiPe2016 life cycle impact assessment method for midpoint environmental impacts and externality categories.
- In Section 2, we give further detail on the harmonization of food items with available LCI data, FAO Food Balance Sheets and *EAT* diet food groups to calculate impacts and externalities of food supply patterns in 2018 and in dietary change scenarios.
- Section 3 presents additional context to our results on the monetarized externalities of national food supplies by comparing with Gross Domestic Product and considering shares of externalities attributed to imported food.
- Section 4 presents results for national, regional and global externalities on a calorie basis.
- Section 5 details the approach we adopted to estimate the greenhouse gas emissions from food production in 2018 and provides a comparison with other literature estimates.
- Section 6 offers the rationale and calculations behind our comparison of the potential GHG reduction from the *VGN* scenario to the reduction required to meet the climate change planetary boundary allocated to food production.
- In Section 7, we provide additional analyses using a more detailed approach to estimating the impacts and externalities associated with imported food.
- Section 8 provides further details regarding parameters used and assumptions in the comparative risk assessment approach we take to estimate health effects from changes in diet composition.
- Section 9 presents additional results on the burden placed on future mineral and fossil resource scarcity by the production of diets.
- Lastly, in Section 10, we discuss the limitations and assumptions of our study followed by some suggestions for potential directions of future work.

Table of Contents

1 Life cycle impact assessment 5

1.1 Life cycle system boundaries for food items 5

1.2 Environmental impact-to-externality damage pathways 7

1.2.1 Environmental impacts contributing to human health burden 7

1.2.2 Environmental impacts contributing to ecosystem quality decline 11

2 Modelling food consumption/supply patterns and calculating impacts 16

2.1 Matching food items with available LCI data to FAO FBS food groups 16

2.2 Calculating impacts from production of domestically produced and imported food 16

2.3 Modelling national food supply quantities with the adoption of the *EAT* diet 16

2.4 Scaling proportion of constituent FAO FBS groups when scaling up supply of plant-based foods in scenarios 17

3 Comparing external costs with Gross Domestic Product 18

4 Externalities per calorie results 19

5 Estimate of greenhouse gas emissions of global food production in 2018 22

6 Meeting the climate change planetary boundary allocated to food production 23

7 Accounting for the countries of origin of imported food 24

8 Parameters and assumptions used to estimate health effects from changes in diet consumption 28

8.1 Relative risk parameters 28

8.2 Theoretical minimum risk exposure levels for dietary risk factors 28

8.3 Estimating DALYs attributable to dietary risk factors and diseases 29

9 Additional results: Impacts on future resource scarcity burden 30

9.1 Cause-and-effect damage pathways: Resource use to economic burden on future resource extraction 30

9.2 Economic implications on resource scarcity from production of 2018 diets and modelled scenarios 32

10 Study limitations, assumptions and directions for future work 35

10.1 The use of average impact intensities 35

10.2 Modelling dietary change scenarios 35

10.3 Monetarizing ecosystem quality 36

10.4 Environmental impact-to-externality damage characterization 36
10.5 Diet composition effects on health and nutrition 37
10.6 Potential directions for future work 37
References 38

This document includes the following supplementary tables:

Supplementary Table 1. Externalities from the production of total food supply (for all countries in each income group classification) as a percentage of total GDP (sum of the GDP of all countries in each income group classification).

Supplementary Table 2. Externalities per calorie (for all countries in each income group classification) and globally.

Supplementary Table 3. Externalities per calorie (for all countries in each geographical region).

Supplementary Table 4. Comparison of estimates (for China) calculated using a more detailed approach for imported food impacts vs. estimates calculating using 'base' approach using export-weighted global average impact factors for imported food.

Supplementary Table 5. Comparison of estimates (for USA) calculated using a more detailed approach for imported food impacts vs. estimates calculating using 'base' approach using export-weighted global average impact factors for imported food.

Supplementary Table 6. Comparison of estimates (for Germany) calculated using a more detailed approach for imported food impacts vs. estimates calculating using 'base' approach using export-weighted global average impact factors for imported food.

Supplementary Table 7. Relative risk (RR) parameters used to calculate population attributable fractions (PAFs) (Equation (9) in the main text) for each dietary risk factor-disease pair considered in the comparative risk assessment.

This document includes the following supplementary figures:

Supplementary Figure 1a-h. General overview of the life cycle stages and activities included in the cradle-to-gate life cycle for (a) whole plant-based food items, (b) meat, (c) eggs, (d) processed plant-based food items, (e) process animal-sourced food items, (f) wild capture fish and seafood, (g) farmed fish and seafood and (h) processed fish oil. Dotted lines represent system boundaries.

Supplementary Figure 2a-g. Cause-and-effect damage pathways modelled in ReCiPe2016 linking midpoint environmental impacts to human health burden.

Supplementary Figure 3a-h. Cause-and-effect damage pathways modelled in ReCiPe2016 linking midpoint environmental impacts to ecosystem quality decline.

Supplementary Figure 4a-b. Damage modelling in ReCiPe2016 of mineral and fossil resource use.

Supplementary Figure 5. Comparison of embedded resource scarcity cost burden vs. reported Final Consumption Expenditure (FCE) on per capita food and non-alcoholic drink consumption in 2018.

Supplementary Figure 6. Economic burden on future fossil and mineral resource scarcity caused by the production of total food supply in all analyzed countries for *BASE* (2018) and modelled dietary change scenarios.

Supplementary Figure 7. Economic burden on future fossil and mineral resource scarcity caused by the production of total food supply in all analyzed countries for *BASE* (2018) and modelled dietary change scenarios.

Other Supplementary Material for this manuscript:

- Supplementary Tables 8 - 13 in Supplementary Data Excel file

1 Life cycle impact assessment

1.1 Life cycle system boundaries for food items

As discussed in the Methods section of the main text, we adopted a cradle-to-gate life cycle approach to estimating the impacts of food items. However, cradle-to-gate encompasses different activities within the system boundaries of food groups. The figures below (Supplementary Figure 1a-h) illustrate the life cycle stages considered for each food group based on the food item processes available in the ecoinvent version 3[1,2], Agri-footprint version 4.0[3] and ESU World Food[4] life cycle inventory (LCI) databases. Using LCI data for each food item, the environmental emissions associated with all inputs, outputs and emissions of activities within the system boundaries are characterised into midpoint impacts (e.g., global warming potential, terrestrial acidification) and endpoint damage (human health burden, ecosystem quality reduction).

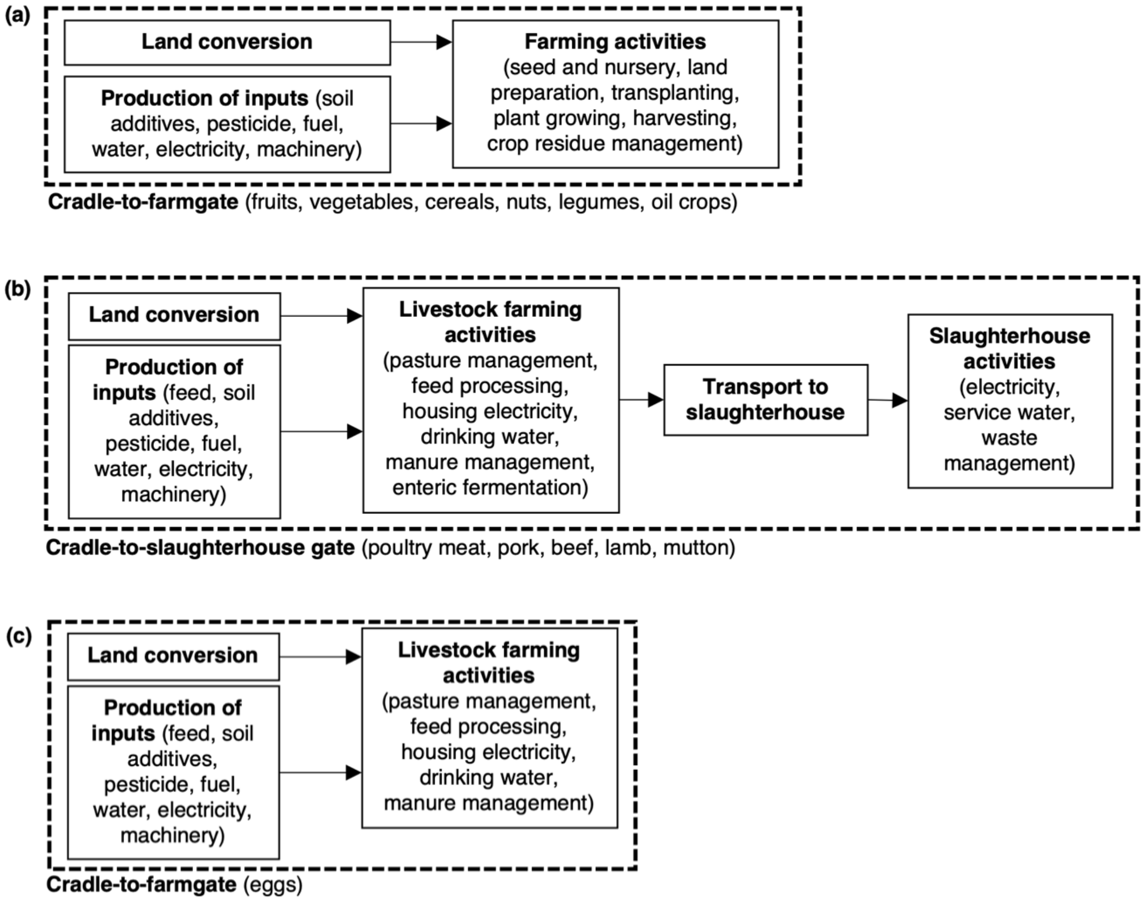

Supplementary Figure 1a-h. General overview of the life cycle stages and activities included in the cradle-to-gate life cycle for (a) whole plant-based food items, (b) meat, (c) eggs. Dotted lines represent system boundaries. (Figure continued on next page)

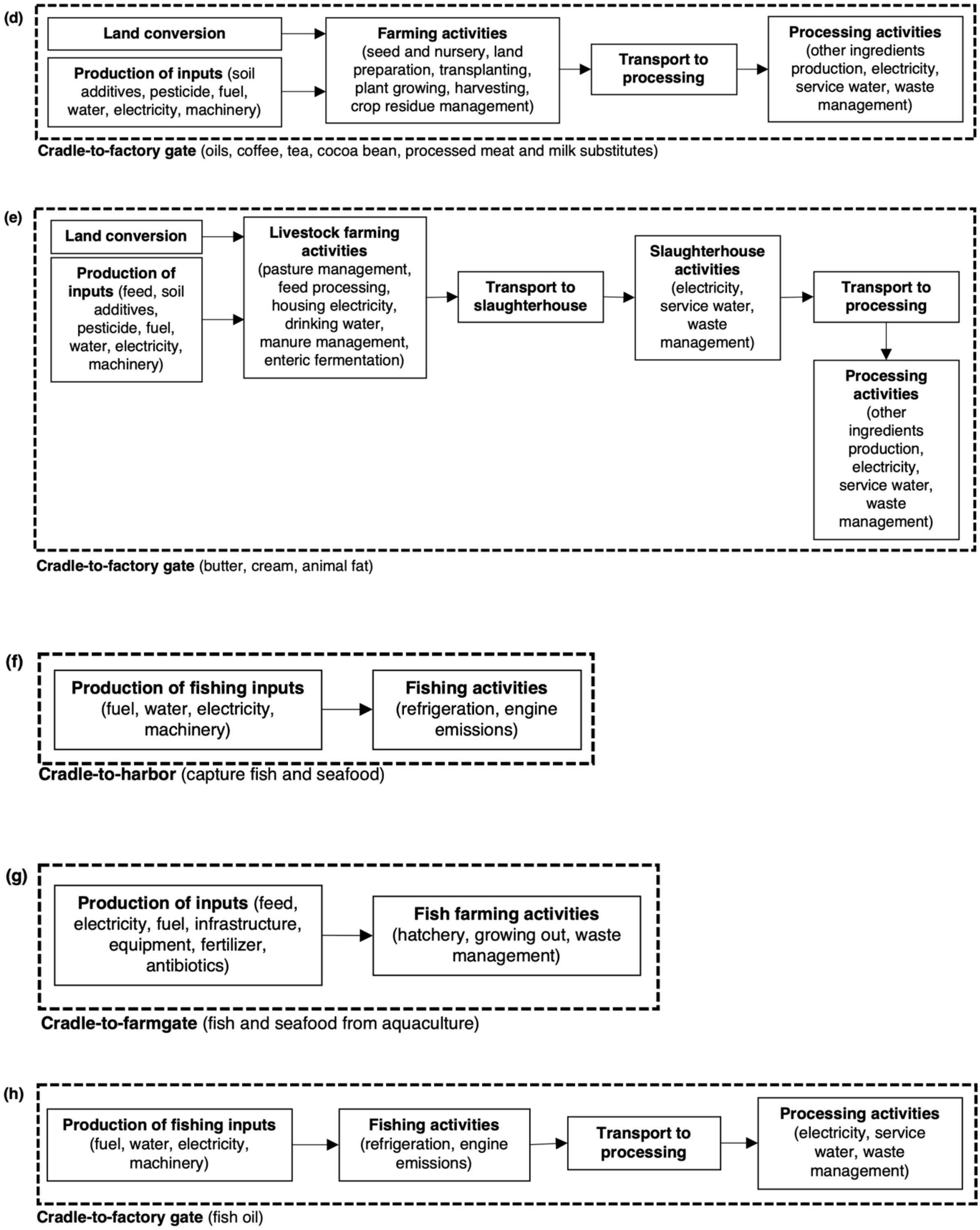

Supplementary Figure 1a-h. General overview of the life cycle stages and activities included in the cradle-to-gate life cycle for (d) processed plant-based food items, (e) process animal-sourced food items, (f) wild capture fish and seafood, (g) farmed fish and seafood and (h) processed fish oil. Dotted lines represent system boundaries.

1.2 Environmental impact-to-externality damage pathways

The life cycle impact assessment (LCIA) method we adopted in this study was the ReCiPe2016 method[5]. The ReCiPe2016 method models cause-and-effect damage pathways between environmental midpoint impacts and endpoint damage (i.e., damage to human health and to ecosystem quality)[6].

All links between environmental impacts and externalities are visualized in Fig. 1 of the main text. This section provides a simplified overview of the mechanisms in which each environmental impact is modelled to ultimately damage human health or ecosystems. Full details on the derivation of all impact-to-externality characterization factors and their underlying damage modelling pathway assumptions can be found in the ReCiPe2016 documentation report[6].

1.2.1 Environmental impacts contributing to human health burden

<u>Water consumption</u>

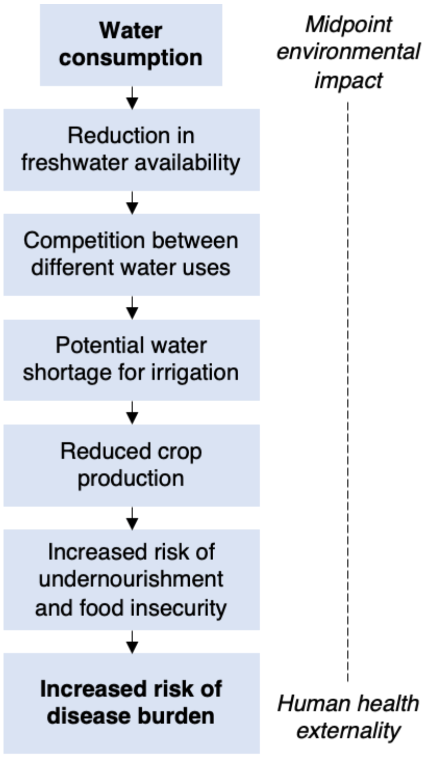

Supplementary Figure 2a. Damage modelling in ReCiPe2016 of water consumption effects on human health burden.

Human toxicity (carcinogenic and non-carcinogenic)

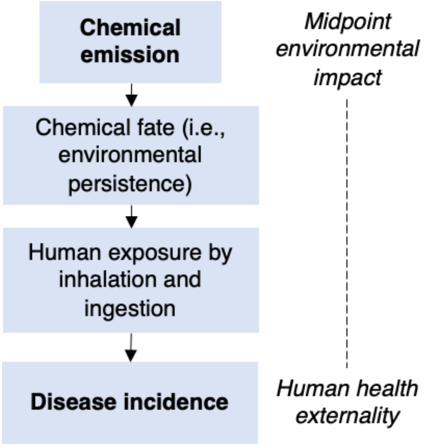

Supplementary Figure 2b. Damage modelling in ReCiPe2016 of human toxicity effects from all chemical emissions.

Fine particulate matter formation

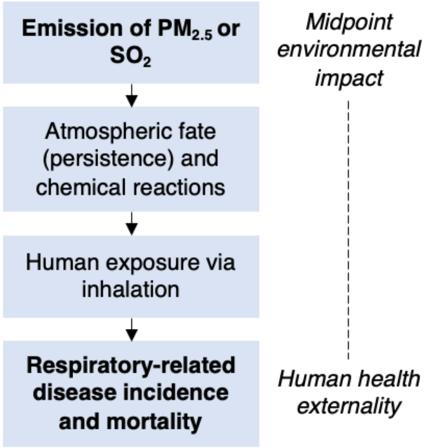

Supplementary Figure 2c. Damage modelling in ReCiPe2016 of human health burden from particulate matter ($PM_{2.5}$ refers to particles with diameters less than 2.6 micrometers) formation from primary aerosol emissions or secondary aerosol SO_2 emissions (precursor to $PM_{2.5}$ formation).

Ozone formation

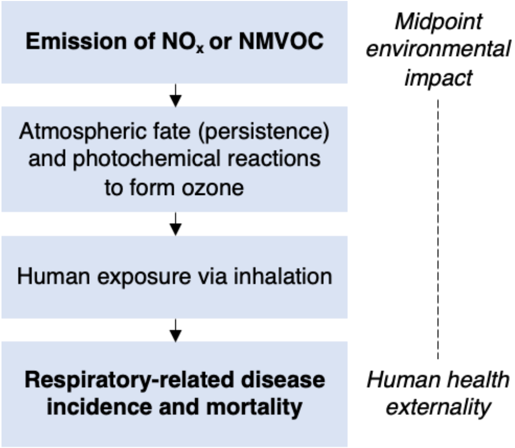

Supplementary Figure 2d. Damage modelling in ReCiPe2016 of human health burden from ozone formation via emission of aerosol NO_x and Non Methane Volatile Organic Compounds (NMVOCs). Chronic exposure to ozone can negatively affect lung function, cause respiratory symptoms, and inflame airways.

Ionizing radiation

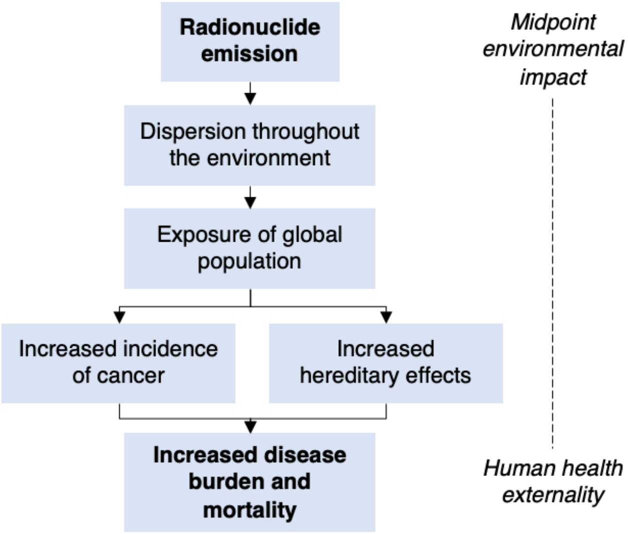

Supplementary Figure 2e. Damage modelling in ReCiPe2016 of human health burden from anthropogenic radionuclide emission (generated from e.g., nuclear fuel mining, processing and waste disposal; coal burning; and phosphate rock extraction).

Stratospheric ozone depletion

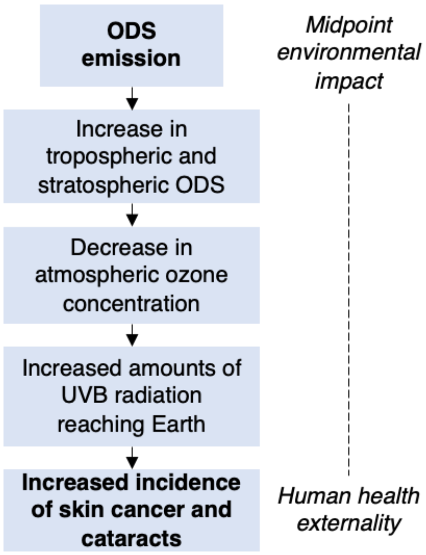

Supplementary Figure 2f. Damage modelling in ReCiPe2016 of human health burden from emission of Ozone Depleting Substances (ODSs).

Global warming

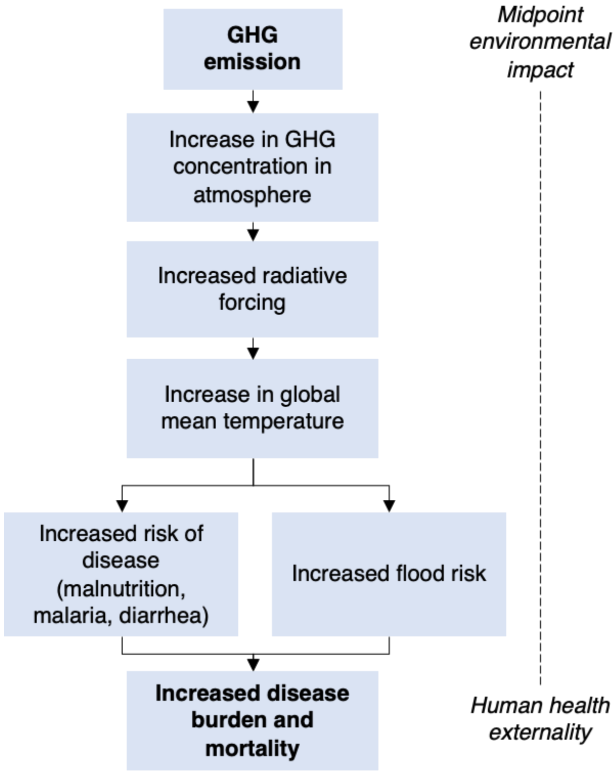

Supplementary Figure 2g. Damage modelling in ReCiPe2016 of human health burden linked to climate change-related disease and flood risk.

1.2.2 Environmental impacts contributing to ecosystem quality decline

Global warming

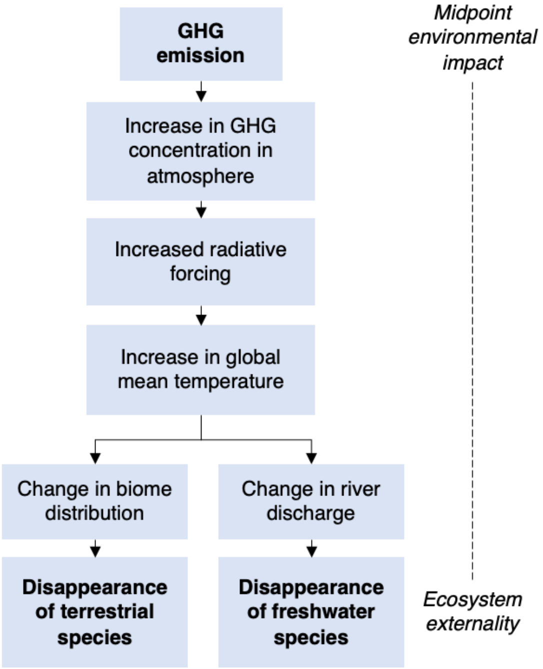

Supplementary Figure 3a. Damage modelling in ReCiPe2016 of ecosystem quality decline (species loss) linked to climate change effects.

Ozone formation

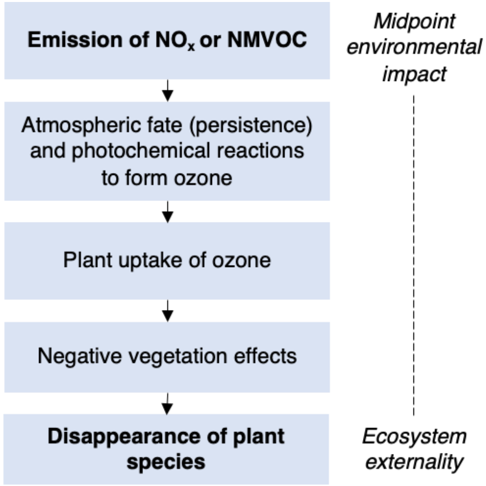

Supplementary Figure 3b. Damage modelling in ReCiPe2016 of ecosystem quality decline (species loss) from ozone formation via plant uptake. Ozone can affect sensitive vegetation by reducing photosynthesis, plant growth and resilience to external factors.

Terrestrial acidification

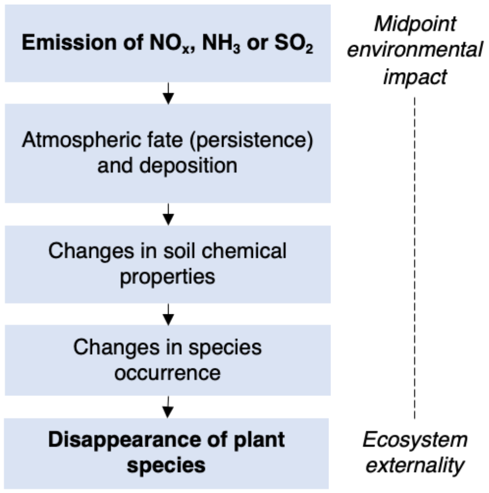

Supplementary Figure 3c. Damage modelling in ReCiPe2016 of ecosystem quality decline (species loss) from ozone formation via plant uptake. Ozone can affect sensitive vegetation by reducing photosynthesis, plant growth and resilience to external factors.

Freshwater eutrophication

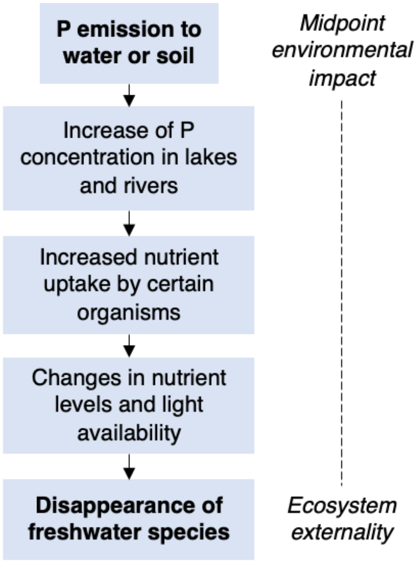

Supplementary Figure 3d. Damage modelling in ReCiPe2016 of freshwater ecosystem quality decline (species loss) from eutrophication, caused by phosphorus emissions.

Marine eutrophication

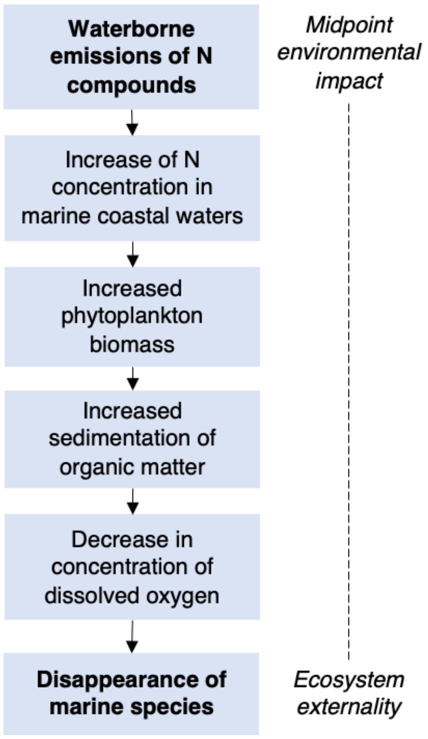

Supplementary Figure 3e. Damage modelling in ReCiPe2016 of marine ecosystem quality decline from eutrophication, caused by waterborne nitrogen compound emissions.

Ecotoxicity (terrestrial, freshwater and marine)

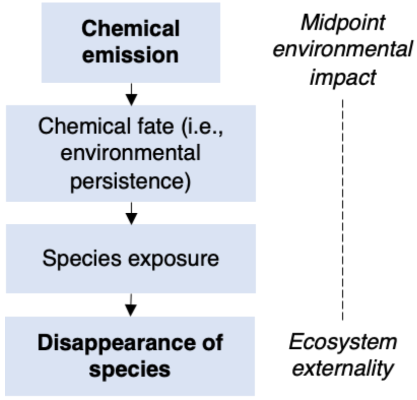

Supplementary Figure 3f. Damage modelling in ReCiPe2016 of terrestrial, freshwater and marine ecosystem toxicity effects from chemical emissions.

Land use

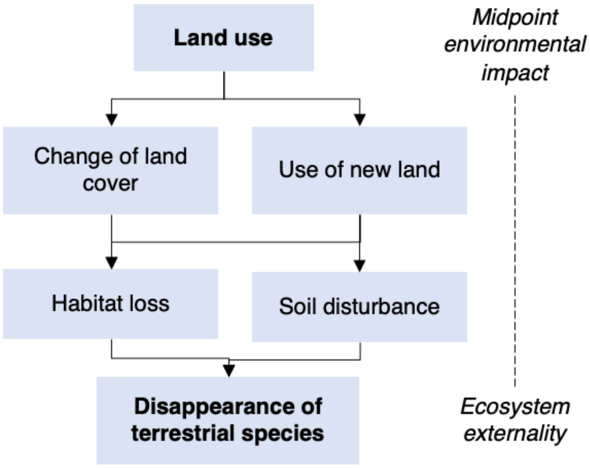

Supplementary Figure 3g. Damage modelling in ReCiPe2016 of land use effects on ecosystem quality.

Water consumption

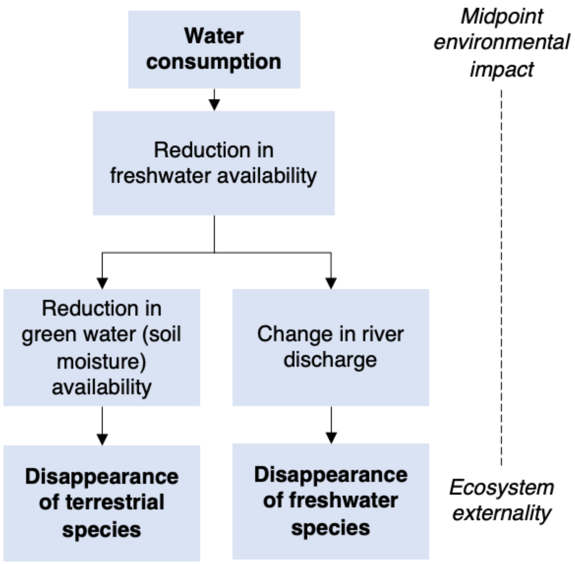

Supplementary Figure 3h. Damage modelling in ReCiPe2016 of land use effects on ecosystem quality.

Note on environmental impacts with regionalized effects on health and/or ecosystems:

The ReCiPe2016 method provides country- or region-specific characterization factors[5,6] (i.e., factors translating midpoint environmental impacts into externality damage) for:

- Fine particulate matter formation
- Ozone formation
- Acidification

- Freshwater eutrophication
- Water use

For remaining environmental impact categories, no spatial differentiation in effects is considered.

In the context of our analysis, however, calculated externality effects should be interpreted on a global level due to our use of global average impact data for many food items and countries (due to the high number of LCI data gaps for country-specific food items).

2 Modelling food consumption/supply patterns and calculating impacts

This section provides further detail on the harmonization of food items with available LCI data, FAO Food Balance Sheets (FBS) and *EAT* diet food groups to calculate impacts and externalities of food supply patterns in 2018 and in dietary change scenarios.

2.1 Matching food items with available LCI data to FAO FBS food groups

As mentioned in the Methods of the main text, each food item from our LCI dataset was matched to the most appropriate FAO FBS food group. For example, for the FAO FBS food group 'onions', we found five 'onion' food item entries in the LCI databases, each corresponding to onion production in different countries. Additionally, FAO FBS food groups are highly aggregated (e.g., one food group is labelled 'Wheat and products'). For simplicity, we generally opted for choosing the primary equivalents of each food group to be representative (e.g., food items matched to 'Wheat and products' are only 'wheat grain' and food items matched to 'Potatoes and products' are only 'potatoes').

2.2 Calculating impacts from production of domestically produced and imported food

For each FAO FBS food group, country-specific impact intensities, if available, were used to estimate the impacts of domestically produced quantities. Global export-weighted average impact intensities were used to calculate impacts from the production of imported food.

For example, taking the FBS food group 'onions' again, the impacts associated with the total supply of 'onions' in China is the sum of impacts from domestically produced supply and impacts from imported supply. Impacts from domestically produced supply is calculated by multiplying the domestically produced supply quantity by the impact intensity of the food item 'onion' which is specific to an onion production process in China (present in the LCI databases). Impacts from imported supply, on the other hand, is calculated by multiplying the imported supply quantity by the export-weighted average of the five country-specific 'onion' food item entries available in the LCI databases.

2.3 Modelling national food supply quantities with the adoption of the *EAT* diet

To model shifts to the *EAT* diet in each country, we first aggregated the 21 food groups–in which the *EAT* diet recommendations are in terms of–into 15 categories (cereals, roots and tubers, vegetables, fruits, dairy, beef and lamb, pork, chicken, eggs, seafood, beans and legumes, soy, nuts and seeds, oils, and sweeteners). For example, the category 'vegetables' is split into *EAT* diet groups 'dark green vegetables', 'red and orange vegetables' and 'other

vegetables'– which cannot be easily matched to the FBS food groups of 'tomatoes and products', 'onions' and 'vegetables, other'.

In a second step, we converted intake amounts into supply quantities using edible portion factors for different food types[9] as the recommended intake values of the *EAT* diet are grams of edible food. Lastly, we scaled the per capita supply quantities of each country to the supply quantities of the *EAT* diet for each food category, maintaining the portion of constituent FBS food groups. For instance–continuing with the example of the food category 'vegetables'–if a country's 2018 baseline supply quantity of 'vegetables' was comprised of 20% 'tomatoes and products', 20% 'onions' and 60% 'vegetables, other', we maintained these proportions of constituent FBS groups when adjusting the quantity of 'vegetables' in the *EAT* scenario.

2.4 Scaling proportion of constituent FAO FBS groups when scaling up supply of plant-based foods in scenarios

We also maintained the proportion of constituent FAO FBS groups in 2018 supply when scaling up the supply quantities of beans, legumes, soybeans, fruits and vegetables to compensate the lost kcal from animal-sourced foods (ASFs). For example, the constituent FBS groups of the beans, legumes and soybeans substitution category are 'beans', 'peas', 'pulses, other and products' and 'soyabeans'. If each constituent FBS group accounted for 25% of the beans, legumes and soybeans supply total in 2018, compensating a loss of 300 kcal/cap/day from removing ASFs would involve a 50 kcal/cap/day increase in each modelled supply quantity of 'beans', 'peas', 'pulses, other and products' and 'soyabeans'.

3 Comparing external costs with Gross Domestic Product

The externalities results can be contextualized considering the countries' GDPs. More specifically, the economic burden and implications of the externalities of food production can be gauged by comparing the monetarized damage to health and ecosystems to national Gross Domestic Product (GDP). Compared to Figure 4 in the main text (where countries are shaded based on absolute monetarized externalities), we observe the opposite trend – i.e., food production externality shares of GDP generally decrease with increasing income (Supplementary Table 1).

Supplementary Table 1. Externalities from the production of total food supply (for all countries in each income group classification) as a percentage of total GDP (sum of the GDP of all countries in each income group classification).

Income group	Monetarized damage from production of food supply (% of total GDP)
Low	99
Lower-middle	56
Upper-middle	27
High	7

4 Externalities per calorie results

In this section, we present results for estimated externalities on a calorie basis. Total or per capita externalities are dependent on the environmental impact intensity (impact per kg or kcal of food) – linked to food type or production practices – and the amount of food supplied (in kg or kcal). By comparing externalities per calorie of countries, income groups and geographical regions, we are therefore able to isolate the influence of diet composition and/or production practices on externality levels (i.e., controlling for amount of food or calories per capita).

The values presented in Supplementary Tables 2 and 3 are total externalities (across all food groups) divided by total calorie supply.

Supplementary Table 2. Externalities per calorie (for all countries in each income group classification) and globally. US$ refers to 2018 US dollars. Values in brackets specify the lower and upper bounds of uncertainty intervals based on: (i) 95% confidence interval values of life cycle impacts of food items (for human health burden, in DALYs/kcal, and ecosystem quality damage impacts, in species loss/kcal); and (ii) 95% confidence interval values of life cycle impacts of food items and low and high estimates of monetarization factors (for total externalities, in US$).

	**Production-caused externalities per calorie supplied**		
Income group	Human health burden (DALYs/kcal)	Ecosystem quality damage (species loss/kcal)	**Total (US$/kcal)**
Low	3.23E-09 (2.38E-09 – 4.41E-09)	2.54E-11 (2.00E-11 – 3.27E-11)	**1.02E-03 (4.43E-04 – 2.35E-03)**
Lower-middle	4.33E-09 (3.05 E-09 – 6.19E-09)	3.08E-11 (2.33E-11 – 4.13E-11)	**1.32E-03 (5.56E-04 – 3.09E-03)**
Upper-middle	7.48E-09 (5.22E-09 – 1.08E-08)	5.02E-11 (3.69E-11 – 6.86E-11)	**2.22E-03 (9.40E-04 – 5.23E-03)**
High	8.50E-09 (6.04E-09 – 1.21E-08)	5.67E-11 (4.17E-11 – 7.78E-11)	**2.52E-03 (1.08E-03 – 5.90E-03)**
**Global**	**6.39E-09 (4.49E-09 – 9.15E-09)**	**4.35E-11 (3.23E-11 – 5.93E-11)**	**1.91E-03 (8.11E-04 – 4.48E-03)**

Externalities per calorie generally increase from low to high income groups (Supplementary Table 2). On a regional level, diets in North America and Oceania have the highest externalities per calorie, while the lowest are associated with diets in Sub-Saharan Africa and South Asia (Supplementary Table 3). We attribute most of the disparity between developed and developing regions to differences in diet composition, rather than variation in production methods of the same foods. We rationalise this attribution through the following:

- Diets in developed countries have greater proportions of animal-sourced foods (ASFs) compared to developing countries where diets are comprised of more cereals, roots, tubers, legumes, nuts and pulses. ASF production generally generates more

environmentally impacts than plant-based foods – e.g., Poore & Nemecek have highlighted how even the 'lowest-impact animal products typically exceed those of vegetable substitutes[10]'.

- Due to the large country and food group gaps in our dataset of food life cycle inventories and impacts (refer to Supplementary Table 13 'Dataset Coverage Summary' in the Supplementary Data file), we cannot safely assume that we have sufficiently captured the variation in production methods of each food group that exists in reality.

Supplementary Table 3. Externalities per calorie (for all countries in each geographical region). US$ refers to 2018 US dollars. Regional grouping of countries can be found in Supplementary Table 12 in Supplementary Data. Underlined results are for regions with top two highest and bottom two lowest total externalities per calorie. Values in brackets specify the lower and upper bounds of uncertainty intervals based on: (i) 95% confidence interval values of life cycle impacts of food items (for human health burden, in DALYs/kcal, and ecosystem quality damage impacts, in species loss/kcal); and (ii) 95% confidence interval values of life cycle impacts of food items and low and high estimates of monetarization factors (for total externalities, in US$).

	**Production-caused externalities per calorie supplied**		
Region	Human health burden (DALYs/kcal)	Ecosystem quality damage (species loss/kcal)	**Total (US$/kcal)**
North America	1.06E-08 (7.48E-09 – 1.51E-08)	7.23E-11 (5.24E-11 – 1.00E-10)	**3.16E-03 (1.34E-03 – 7.50E-03)**
Europe	7.21E-09 (5.20E-09 – 1.01E-08)	4.87E-11 (3.65E-11 – 6.57E-11)	**2.15E-03 (9.35E-04 – 4.97E-03)**
Oceania	1.17E-08 (8.26E-09 – 1.67E-08)	7.96E-11 (5.83E-11 – 1.09E-10)	**3.49E-03 (1.49E-03 – 8.23E-03)**
West Asia	5.33E-09 (3.76E-09 – 7.51E-09)	3.73E-11 (2.77E-11 – 5.05E-11)	**1.61E-03 (6.82E-04 – 3.76E-03)**
East Asia	7.45E-09 (5.13E-09 – 1.09E-08)	4.78E-11 (3.47E-11 – 6.63E-11)	**2.17E-03 (9.15E-04 – 5.15E-03)**
Latin America and the Caribbean	8.43E-09 (6.06E-09 – 1.18E-08)	6.02E-11 (4.53E-11 – 8.06E-11)	**2.57E-03 (1.10E-03 – 5.97E-03)**
Sub-Saharan Africa	3.71E-09 (2.69E-09 – 5.69E-09)	2.83E-11 (2.19E-11 – 3.71E-11)	**1.16E-03 (4.97E-04 – 2.69E-03)**
North Africa	4.58E-09 (3.13E-09 – 6.57E-09)	3.30E-11 (2.42E-11 – 4.50E-11)	**1.40E-03 (5.73E-04 – 3.33E-03)**
South-east Asia	6.56E-09 (4.66E-09 – 9.38E-09)	4.21E-11 (3.07E-11 – 5.84E-11)	**1.91E-03 (8.28E-04 – 4.49E-03)**
South Asia	3.88E-09 (2.69E-09 – 5.57E-09)	2.82E-11 (2.16E-11 – 3.75E-11)	**1.19E-03 (4.95E-04 – 2.79E-03)**
Central Asia	8.40E-09 (5.88E-09 – 1.20E-08)	6.05E-11 (4.47E-11 – 8.23E-11)	**2.57E-03 (1.07E-03 – 6.08E-03)**

More specifically, the countries with the top three highest total externalities per calorie (and their income group classification):

1. Australia (H, Oceania) [US$ 0.0036 (0.0015-0.0084) per calorie]
2. Thailand (UM, South-east Asia) [US$ 0.0035 (0.0015-0.0084) per calorie]
3. Switzerland (H, Europe) [US$ 0.0033 (0.0014-0.0079) per calorie]

The bottom three countries with the lowest total externalities per calorie:

99. Nigeria (LM, Sub-Saharan Africa) [US$ 0.0009 (0.0004-0.0021) per calorie]
100. Ethiopia (L, Sub-Saharan Africa) [US$ 0.0009 (0.0004-0.0019) per calorie]
101. Ghana (LM, Sub-Saharan Africa) [US$ 0.0008 (0.0003-0.0019) per calorie]

5 Estimate of greenhouse gas emissions of global food production in 2018

We followed a ‘bottom-up’ approach to estimate the greenhouse gas (GHG) emissions from food production. As outlined in the Methods section of the main text, we first multiplied the amount of each food item that is supplied in every country by its country-specific or global average global warming potential intensity (kgCO_2e per kg of food, derived from LCI databases). The total GHG emissions associated with each country’s food supply were then estimated by summing GHG emissions across all 689 food items. The global total of GHG emissions from food production was estimated by adding all GHG estimates across all countries included in our analysis.

Following this approach, we estimate 9.1 (6.4 – 12.7) GtCO_2e of GHG emissions associated with the production of food supplied in 101 analysed countries in 2018. Our estimate is comparable to other literature estimates; more specifically, the upper bound of our 95% confidence interval is similar to other literature estimates for food production and processing of 13.7 GtCO_2e in 2015[11] and 11.9 GtCO_2e in 2010[10]. This slight mismatch might be a result of failing to cover the entire global population in our analysis (i.e., only 91% of the global population in 2018). Moreover, assumptions were made regarding the GHG emissions intensities due to data gaps in our LCI dataset of food items. GHG intensities of a food item typically vary across countries (due to different production methods), but in some cases in our analysis, we assumed global average values for countries lacking data for specific food items. We sought to address the uncertainty arising from such data gaps through Monte Carlo simulations providing lower and upper bound values of the 95% confidence interval (further details are discussed in the Methods section of the main text).

6 Meeting the climate change planetary boundary allocated to food production

In the Results section of the main text, we compare the potential savings in GHG emissions achievable by the *VGN* dietary change scenario to the reduction required from 2018 levels to meet the food production boundary of the planetary safe operating space of climate change, given by the *EAT-Lancet* Commission[24].

To estimate the percentage of the GHG emission reduction required to meet the climate change boundary for food production that the *VGN* scenario could achieve, we undertook the following calculation. As discussed in Section 5 in this Supplementary Information document, we estimated that the total GHG emissions from producing the food supplied in all countries in our analysis in 2018 (i.e., *BASE* scenario) are 9.1 (6.4-12.7) GtCO_2e. The share of the planetary safe operating space for climate change allocated to food production is determined by the *EAT-Lancet* Commission to be 5 GtCO_2e per year. The reduction from our 2018 estimate to meet the climate change boundary for food production is, therefore, 4.1 (1.4-7.7) GtCO_2e. We then compared this required reduction to the savings we estimate to be achievable by the *VGN* dietary change scenario [4.5 (3.9-5.8) GtCO_2e]. By dividing the potential savings from the *VGN* scenario by the reduction required to meet the climate change planetary boundary food production, we estimated that *VGN* savings represent 110% of the reduction needed (using point estimates; 280% using lower bound estimates and 75% using upper bound estimates).

7 Accounting for the countries of origin of imported food

In our analysis, we have used export-weighted global average impact factors (impact/kg of food) to estimate the impacts and externalities from imported food. We opted for this approach because of the major gaps in our food life cycle inventory (LCI) dataset across countries and food groups (refer to Supplementary Table 13 'Dataset Coverage Summary' in the Supplementary Data file). We acknowledge, however, that this approach masks variability across the production practices of exporting countries, and accounting for the countries of origin of imported food (i.e., using country-specific impact factors in calculating impacts of imported food) may modify our presented results.

We chose 'test' case countries to compare results from our 'base' approach to those estimated via a more detailed approach to calculating impacts from imported food quantities. This detailed approach involved using impact factors specific to countries of origin for imported food, instead of export-weighted global averages. The aim of this comparison was to check the extent to which using country-specific impact factors for imported food shares affects our final estimates of externalities (i.e., externalities embedded in diets of countries considering domestically produced and imported food).

We chose to do a detailed analysis on the impacts of imported food for countries with the highest quantities of imported food in 2018. Based on reported import quantities in the FAO Food Balance Sheets (FBS), the top three importing countries were China (mainland), USA and Germany.

For each country, we referred to the FAOSTAT Detailed Trade Matrices (TMs) for quantities of imports and their countries of origin.

The steps in the detailed approach we took to estimating the impacts from imported food shares are as follows:

i. Identifying countries of origin: For each FAO FBS food group (e.g., 'Wheat and products' or 'Bananas'), we used the FAOSTAT TMs to calculate the proportion of food imports from each country of origin.

ii. Isolating top five countries of origin for each food group: We then isolated the top five countries of origin for each food group (i.e., top five countries of origin providing highest imported food quantities). This was done because of large country and food group gaps in our dataset of food life cycle inventories (LCIs) (refer to Supplementary Table 13 'Dataset Coverage Summary') – i.e., it would not have been possible to match all countries of origin with their country-specific LCI and impact factor across all food groups. Nevertheless, we found that the top five countries of origin account for the majority of total imports across all FBS food groups and test countries.

iii. Estimating quantities of food available for human consumption imported from top five countries of origin: Proportion of imports for all top five countries of origin were then used to estimate their relative contributions to domestic food supply – i.e., proportions of each country of origin were multiplied by the quantity of imported food assumed to be available for human consumption (estimated share from imports in the 'Food' element in the FBS).

iv. Estimating externality impacts from imported food:
   a. For top five countries of origin:
      - If the country- and food-specific LCI is available in our dataset, the estimated food quantity from country of origin was multiplied by its matching impact factor.
      - If the country- and food-specific LCI is not available, the estimated quantity from country of origin was multiplied by the global average impact factor for each food group.
      - Note: Across all three test countries, we were only able to match ~20% of required country-specific impact factors.
   b. For remaining imported food quantities: Remaining quantity of estimated imported food available for human consumption was multiplied by global average impact factor for each food group.

Note: Fish and seafood groups are not covered in the FAOSTAT TMs. Fish and seafood import flows were therefore not included in this detailed approach. In any case, if countries of origin for fish and seafood import flows were available, global average impact factors would likely have to be used due to the limited coverage of fish/seafood items in our LCI dataset (refer to Supplementary Table 13 'Dataset Coverage Summary').

Compared to the results from our 'base' approach, the above detailed approach yielded the following results for each test country (Supplementary Tables 4, 5 and 6):

Major importing country 1: China, mainland

- Top five countries of origin account for % of imported food shares across FBS groups: 65 – 100%
- Percentage (%) of required China-specific LCI which were available in our dataset: 20% (69 of 344)

Supplementary Table 4. Comparison of estimates (for China, mainland) calculated using a more detailed approach for imported food impacts vs. estimates calculating using 'base' approach using export-weighted global average impact factors for imported food.

**Externalities associated with available food supply (domestic production + imported shares)**	Lower bound of CI from **base approach***	Estimate from **base** approach	Estimate from **detailed TM approach**	Upper bound of CI from **base approach***	Is detailed approach estimate within CI of base approach?
Human health damage (DALYs)	8.25E+06	1.20E+07	1.22E+07	1.75E+07	Yes
Ecosystem quality damage (species loss this century)	5.61E+04	7.74E+04	7.88E+04	1.07E+05	Yes

*Confidence interval (CI) values are from uncertainty analyses accounting for uncertainty associated with LCI data and monetarization factors.

Major importing country 2: USA

- Top five countries of origin account for % of imported food shares across FBS groups: 54 – 100%
- Percentage (%) of required USA-specific LCI which were available in our dataset: 22% (81 of 365)

Supplementary Table 5. Comparison of estimates (for USA) calculated using a more detailed approach for imported food impacts vs. estimates calculating using 'base' approach using export-weighted global average impact factors for imported food.

**Externalities associated with available food supply (domestic production + imported shares)**	Lower bound of CI from **base approach***	Estimate from **base** approach	Estimate from **detailed TM approach**	Upper bound of CI from **base approach***	Is detailed approach estimate within CI of base approach?
Human health damage (DALYs)	3.30E+06	4.67E+06	4.85E+06	6.66E+06	Yes
Ecosystem quality damage (species loss this century)	2.30E+04	3.19E+04	3.31E+04	4.43E+04	Yes

*Confidence interval (CI) values are from uncertainty analyses accounting for uncertainty associated with LCI data and monetarization factors.

Major importing country 3: Germany

- Top five countries of origin account for % of imported food shares across FBS groups: 54 – 99%
- Percentage (%) of required Germany-specific LCI which were available in our dataset: 26% (94 of 364)

Supplementary Table 6. Comparison of estimates (for Germany) calculated using a more detailed approach for imported food impacts vs. estimates calculating using 'base' approach using export-weighted global average impact factors for imported food.

**Externalities associated with available food supply (domestic production + imported shares)**	Lower bound of CI from **base approach***	Estimate from **base** approach	Estimate from **detailed TM approach**	Upper bound of CI from **base approach***	Is detailed approach estimate within CI of base approach?
Human health damage (DALYs)	4.86E+05	6.66E+05	7.28E+05	9.26E+05	Yes
Ecosystem quality damage (species loss this century)	3.36E+03	4.48E+03	4.87E+03	6.03E+03	Yes

*Confidence interval (CI) values are from uncertainty analyses accounting for uncertainty associated with LCI data and monetarization factors.

Generally, using impact factors specific to countries of origin for imported food in all three test countries slightly increased the external costs embedded in their diets. This trend indicates that test countries import food from countries with more environmentally damaging agricultural production practices than the global average.

For all three test countries, however, the new estimates of total externalities associated with available food using the detailed approach for import shares are within the base approach confidence intervals. Our uncertainty analyses on base approach estimates (refer to Methods section of main text for further details) can therefore be assumed to capture variability within the dataset we have available to us arising from adjusting impacts of imported food to their respective countries of origin. Through this supplementary analysis, we seek to demonstrate that accounting for the countries of origin for imported food would not offer significant improvements due to the limited coverage of currently available data.

8 Parameters and assumptions used to estimate health effects from changes in diet consumption

8.1 Relative risk parameters

Supplementary Table 7. Relative risk (RR) parameters used to calculate population attributable fractions (PAFs) (Equation (9) in the main text) for each dietary risk factor-disease pair considered in the comparative risk assessment.

Dietary risk factor	Disease endpoint	Unit	RR (mean)	RR (low 95% C.I. value)	RR (high 95% C.I. value)
Low intake of fruits	Coronary heart disease[25]	100 g/day	0.95	0.92	0.99
	Stroke[25]		0.77	0.70	0.84
	Cancer[25]		0.94	0.91	0.97
Low intake of vegetables	Coronary heart disease[25]	100 g/day	0.84	0.80	0.88
	Cancer[25]		0.93	0.91	0.95
Low intake of legumes	Coronary heart disease[26]	57 g/day	0.86	0.78	0.94
High intake of red meat	Coronary heart disease[27]	100 g/day	1.15	1.08	1.23
	Stroke[27]		1.12	1.06	1.17
	Colorectal cancer[28]		1.12	1.06	1.19
	Type-II diabetes[29]		1.17	1.08	1.26

Relative risk parameters quantify the effect of changes in dietary risk factors on disease endpoints. For example, the mean relative risk parameter for red meat intake and stroke is 1.12 with units of 100 g/day meaning that the dose-response analysis of primary studies found that the risk of stroke increases by 12% for every 100g/day increase in red meat intake.

Other major dietary intake risk factors such as intake of processed meat and whole grains were not considered as we did not consider further levels of processing within FAO FBS food groups – e.g., we did not consider the proportion of beef/pork/chicken supply or intake that is processed, or of cereals that is in whole grain or processed form.

8.2 Theoretical minimum risk exposure levels for dietary risk factors

The theoretical minimum risk exposure level (TMREL) values used for each dietary risk factor (adopted as the alternative ideal exposure scenario) were adopted from Micha et al.[30] with

modifications for vegetables and red meat in line with the approach taken in the Global Nutrition Report 2021[31,32].

- Fruits: 300 g/day
- Vegetables: 500 g/day
- Legumes: 100 g/day
- Red meat: 0 g/day

8.3 Estimating DALYs attributable to dietary risk factors and diseases

To estimate the number of DALYs attributable to the dietary risks we considered, we multiplied our PAF estimates with the reported number of disease-specific DALYs in 2018 for each country, taken from the Global Burden of Disease Study database[33]. As only chronic diseases are included in our analysis, we consider the incidence of these diseases in adults aged 20+.

9 Additional results: Impacts on future resource scarcity burden

This section presents additional results regarding the economic implications of mineral and fossil resource use, calculated by the ReCiPe2016[6] method. These results are not included in the main set of results (focusing on the external costs of diets) as the economic burden on future resource scarcity and extraction is implicitly embedded in the prices of food. Nevertheless, the impacts of food production on future resource scarcity and their economic implications future generations are important to consider. The additional results presented in this section shed light on the extent to which dietary changes away from ASFs can reduce the burden on future resource scarcity.

9.1 Cause-and-effect damage pathways: Resource use to economic burden on future resource extraction

The impacts on resource scarcity modelled in the ReCiPe2016[6] method refer to the economic implications of increased resource depletion, expressed as the estimated increase in future operating costs caused by current extraction of mineral and fossil resources.

<u>Mineral resource scarcity effects</u>

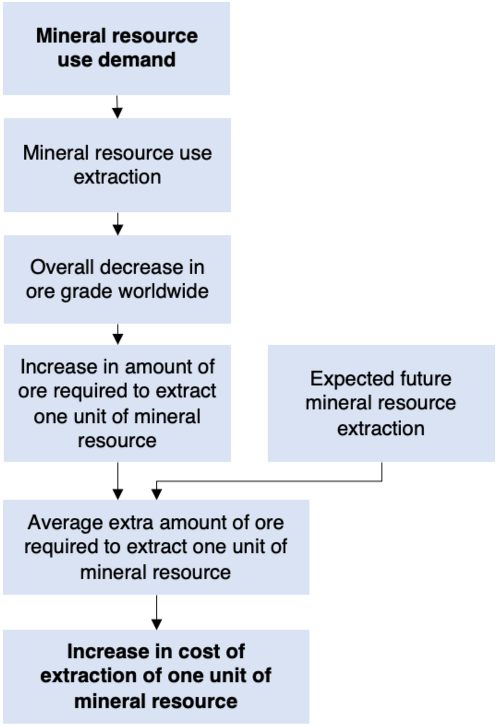

Supplementary Figure 4a. Damage modelling in ReCiPe2016 of mineral resource use.

For mineral resource use, characterization modelling in ReCiPe2016 assumes that mineral resource extraction–demanded by the production of a food item–lead to an overall decrease

in ore grade which, alongside expected future extraction for all other activities, increases the amount of ore required to extract the mineral in the future. To estimate the decrease in ore grade as a result of additional mineral extraction, a log-logistic regression is used to represent the relationship between ore grade and cumulative mineral extraction[6,7]. This additional ore requirement can then be used to estimate the economic burden that a unit of current mineral extraction places on future extraction requirements based on a log-logistic regression curve between operating costs and cumulative mineral extraction and the assumption that mining sites with lower costs are the first to be explored[6,7].

Fossil resource scarcity effects

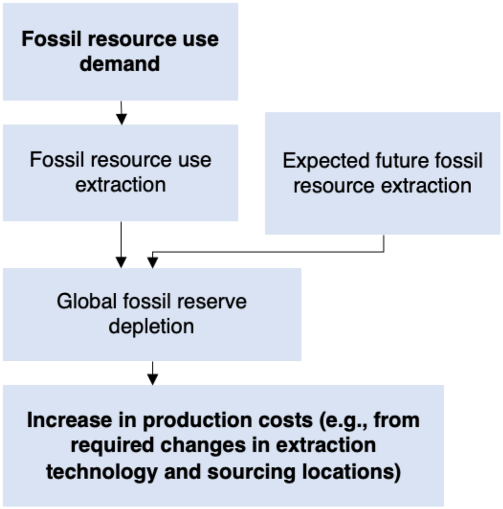

Supplementary Figure 4b. Damage modelling in ReCiPe2016 of fossil resource use.

Similarly, for fossil resource extraction, an increase in fossil fuel extraction–combined with expected future extraction for all activities–is assumed to increase future operating costs due to required changes in production methods and/or sourcing from more cost-intensive locations. A log-linear relationship is assumed between the production cost of a fossil resource and the cumulative extraction of that resource[6,8].

9.2 Economic implications on resource scarcity from production of 2018 diets and modelled scenarios

As endpoint impacts on resource availability calculated by the ReCiPe2016 LCIA characterization method are expressed in terms of required additional costs for future resource extraction in 2013 US$, we converted values to 2018 US$ equivalents.

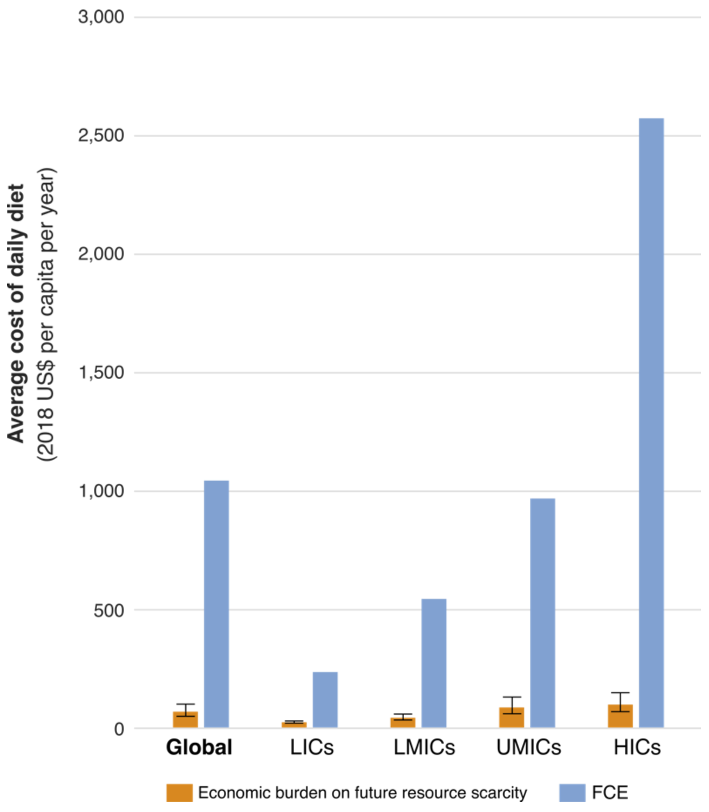

Supplementary Figure 5. External cost of the production of consumed food and non-alcoholic drink on mineral and fossil resource scarcity compared to reported Final Consumption Expenditure (FCE). Bars present the mean cost values of diets globally (Global), in low-income (LICs), lower-middle-income (LMICs), upper-middle-income (UMICs) and high-income (HICs) countries. Error bars indicate lower and upper bounds of the uncertainty range on the average external costs of diets, based on the 95% confidence interval values of life cycle impacts of food items (n=1000 Monte Carlo simulation runs). Error bars are not provided on bars showing average FCE as confidence intervals on expenditure values were not available.

We estimate that the economic burden on future resource scarcity that was embedded in the cost of average diets in 2018 was only a relatively small portion of its cost to consumers (Supplementary Fig. 5). We also identified a trend across income group classification as the cost burden of fossil and mineral resource use in the production of the average diet was found to increase from low to high income. In other words, the types of food generally consumed in higher income countries are more resource use intensive (mostly fossil, as shown in Supplementary Fig. 6), thus placing a greater burden on the extraction of these resources for future generations.

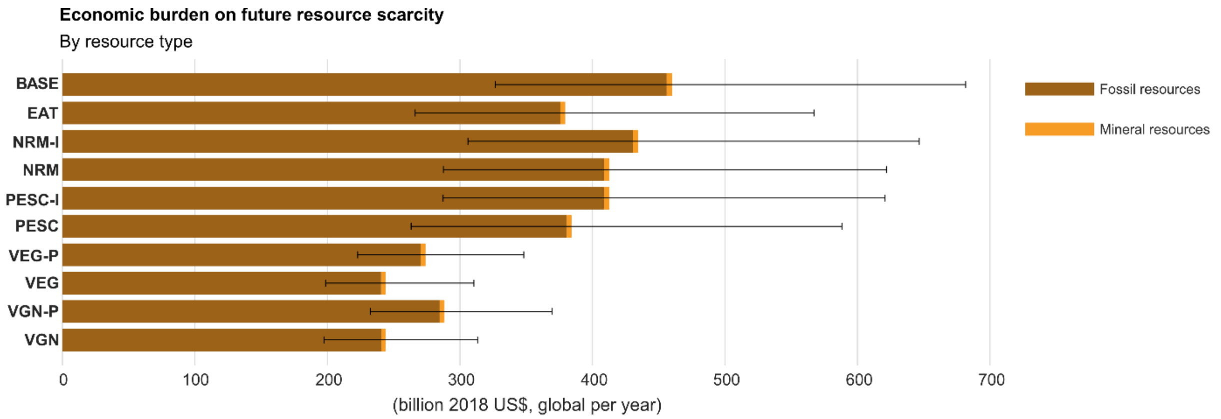

Supplementary Figure 6. Economic burden on future fossil and mineral resource scarcity caused by the production of total food supply in all analyzed countries for *BASE* (2018) and modelled dietary change scenarios. Bars are broken down by resource type (fossil vs. mineral). Error bars indicate lower and upper bounds of the uncertainty range on the total global costs to resource burden scarcity of each scenario based on the 95% confidence interval values of life cycle impacts of food items (n=1000 Monte Carlo simulation runs).

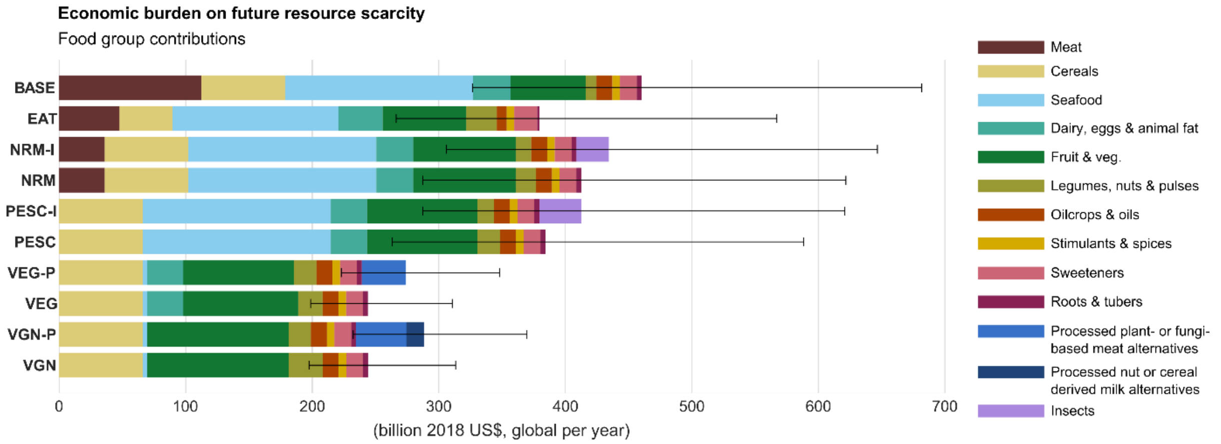

Supplementary Figure 7. Economic burden on future fossil and mineral resource scarcity caused by the production of total food supply in all analyzed countries for *BASE* (2018) and modelled dietary change scenarios. Bars are broken down by contribution from the production of each food group. Constituent food items of each food group are listed in Supplementary Table 9 in the Supplementary Data. Error bars indicate lower and upper bounds of the uncertainty range on the total global costs to resource burden scarcity of each scenario based on the 95% confidence interval values of life cycle impacts of food items (n=1000 Monte Carlo simulation runs).

Increases in future operating costs of resource extraction could most effectively be avoided by lowering both seafood and meat intake (Supplementary Fig. 7) – i.e., the most pronounced savings correspond to moving from *BASE* to *VEG*. These savings correspond mainly to prevented increases in future fossil resource extraction operating costs caused by more depleted fossil reserves (Supplementary Fig. 6). Lowering meat consumption is one way to reduce fossil fuel demand for fertilizer production (for feed crop farming), as well as for energy use in farm machinery and/or confinement operations. Reducing seafood intake, on the other hand, would lessen fossil resource depletion as mechanized fishing activities on motorized fishing vessels largely rely on fossil fuel inputs, while aquaculture operations–e.g., pumping, heating/cooling, wastewater treatment–are mainly powered by fossil-based electricity and fuel.

The notable increase in fossil resource use intensity of processed plant-based food production compared to whole plant-based foods is also apparent in Supplementary Fig. 7 – i.e., comparing the resource scarcity cost burden of *VEG* to *VEG-P* and *VGN* to *VGN-P*. Similarly, the production of insects has notable contributions to the fossil resource burden of the *NRM-I* and *PESC-I* diets. These highlighted contributions from processed plant-based food analogues and insects underline the importance of powering production using renewable energy sources, and of insect feed not dependent on fossil-based inputs.

10 Study limitations, assumptions and directions for future work

This study has shed light on the extent and nature of the external production-related costs embedded in global dietary patterns, as well as on the potential for dietary change to mitigate such costs. More detailed interpretation of the findings of this study, however, are subject to some limitations and assumptions.

10.1 The use of average impact intensities

When estimating impacts caused by domestic production, we assumed average global impact intensity values, as described in Equation (4) in the main text, where country-specific LCI data were not available for a specific food group. In cases where country-specific LCI data were available for a certain food, we applied country average impact values [Equation (3)]. We acknowledge that production methods vary widely across countries and using country or global averages mask such variation and lead to uncertainties in our national and global food supply impact estimates. Additionally, through the use of country average impact values, we may also be masking patterns in the difference in impacts between producers for the domestic vs. international markets – e.g., export-oriented producers, in reality, may have more environmentally impactful practices than producers for the domestic market.

As also discussed in the Methods section of the main text, we sought to address these uncertainties arising from data gaps through Monte Carlo simulations which provide the 95% confidence interval values for food item impact intensities.

Due to large data gaps in our LCI dataset of food groups for each country, we used export-weighted global average impact values to estimate the environmental impacts associated with the production of imported food. In turn, our results only detail the magnitude of damage caused by each country's food supply but not its geographical distribution. Therefore, externalities associated with food consumed in a country are not necessarily linked to impacts taking place in that country and its costs may not directly affect its economy and citizens. Where externalities are 'felt' depends on where supplied food is originally produced (i.e., whether food is imported or domestically produced) and whether the cause-and-effect pathways of midpoint impacts have localised or global effects on endpoint areas of protection. For example, contributions to global warming through emissions from food production in Europe would be more closely linked to human health burden in climate-vulnerable regions. The results presented from this analysis should, therefore, be interpreted from a global perspective – i.e., the externalities we found to be embedded in dietary patterns are assumed to affect overall global ecological and socio- economic systems.

10.2 Modelling dietary change scenarios

We used the national per capita food supply as a proxy for the average diet of each country. Hence, we assumed that the relative proportions of imported and domestically produced food

remains constant when scaling national supply down to the average per capita diet[34]. This ratio was also maintained when scaling national supply quantities to model dietary change scenarios. We acknowledge that, in reality, however, the ratio of imported to domestically produced food can vary significantly across individuals due to personal preference, income, where food is purchased, etc.

We assumed that the global economy could be adjusted to supply and deliver the necessary amount of food items in each dietary scenario. We did not consider capacity constraints relating to agricultural production capacity (e.g., land and water availability, soil and climate suitability) or supply chain structures. For example, a widespread transition to plant-based diets which substitute ASFs with processed plant-based foods (such as mycoprotein) may not be currently feasible given current production capacities.

Regarding the dietary change scenarios we modelled, we made assumptions on which foods, and in what proportions, would replace ASFs (outlined in Table 1 of the main text). We acknowledge that there are innumerable ways in which individuals can choose to adopt a less ASF-dependent diet and are likely to be influenced by many cultural and socio-economic factors. However, the drivers of differences in dietary change and behaviour across countries and regions are not within the scope of this study. Accordingly, we implemented sensible ASF substitution rules which generally reflect transitions to pescatarian, vegetarian and vegan diets, based on previously adopted substitution rules in scientific literature[35]. Furthermore, we also assumed that edible insects (*NRM-I, PESC-I* scenarios) and processed meat and milk alternatives (*VEG-P, VGN-P* scenarios) could be widely accepted and readily consumed worldwide despite current cultural, economic and production constraint barriers.

10.3 Monetarizing ecosystem quality

Given the complexity and controversial nature of expressing damage to ecosystems in monetary value, there is a large uncertainty range between the lower and upper values of the monetarization factor for species loss based on the work by Weidema[38,39]. In turn, the ranges between our lower and upper estimates for monetarized ecosystem damage from food supplies are also significant. However, as acknowledged by Weidema, the currently proposed monetarization factor serves only as a proxy value until better estimates can be derived directly from choice modelling[38,39]. In the meantime, this current proxy value allows studies such as ours to provide an initial gauge of the economic implications of ecosystem damage caused by anthropogenic activities while demonstrating the advantages of translating ecosystem damage into monetary value.

10.4 Environmental impact-to-externality damage characterization

The environmental impact-to-externality damage pathways in ReCiPe2016[6] (summarized in Section 1.2 of this document) are based on high-level modelling assumptions and are subject to varying levels of uncertainty. However, uncertainty ranges on the characterization factors

converting environmental impacts to their externality damage contribution are not currently available in the ReCiPe2016 methodology (e.g., uncertainty on the number of Disability-Adjusted Life Years caused by 1kg of CO_2e emitted). Additionally, as acknowledged by the ReCiPe2016 method developers[5], not all damage pathways have been modelled and other important pathways should be included in future updates of the method – e.g., change in the risk of infectious diseases from global warming or adverse health effects from pesticides on food.

10.5 Diet composition effects on health and nutrition

When estimating the health effects of changes in food consumption in our dietary scenarios, we consider only diet-related chronic disease (coronary heart disease, stroke, cancer and type-II diabetes) due to the availability of dietary intake -disease risk evidence and parameters. As such, we do not consider the potential effects of diet scenarios on incidence of wasting, stunting, underweight and vitamin/mineral deficiencies related to undernutrition. This exclusion could be critical for LICs and LMICs as removing ASFs from diets in these regions may lead to an increase in DALYs related to undernutrition. Our results are, therefore, likely overestimating the positive consumption-related health effects from reducing ASF intake for lower income regions. Future dietary change scenario assessments seeking to build on the approach we adopt in this analysis could consider undernutrition-related health externalities to offer 'fuller' accounting of the potential consequences of dietary change.

Due to the relative novelty of processed plant-based ASF substitutes and insects, to the best of our knowledge there is little to no available epidemiological evidence yet studying the relationship between the intake of these food groups to changes in risk of chronic disease. Consequently, we were not able to capture any potential consumption-related health effects from dietary change scenarios including processed plant-based ASF substitutes and insects (*NRM-I, VEG-P, VGN-P*).

10.6 Potential directions for future work

Concerning future research directions, future work could investigate the regionalization of environmental impact and externalities, complex system dynamics and feedbacks between food consumption, production and markets, as well as consumption-related impacts. The latter effects could be studied following a consequential LCA framework and/or using Integrated Assessment Models (IAMs). Additionally, other demand-side mitigation techniques could be considered alongside dietary change, such as healthy calorie intake adjustments and food loss and waste reductions.

References

1. Wernet, G. *et al.* The ecoinvent database version 3 (part I): overview and methodology. *Int J Life Cycle Assess* **21**, 1218–1230 (2016).

2. Ecoinvent. Ecoinvent database (Version 3.5) [Cut-off system model]. Preprint at (2018).

3. Blonk Consultants. Agri-footprint Database (Version 4.0) [Economic allocation]. Preprint at (2017).

4. ESU-services Ltd. ESU World Food LCA Database. Preprint at http://esu-services.ch/data/fooddata/ (2020).

5. Huijbregts, M. A. J. *et al.* ReCiPe2016: a harmonised life cycle impact assessment method at midpoint and endpoint level. *International Journal of Life Cycle Assessment* **22**, 138–147 (2017).

6. Huijbregts, M. A. J. *et al.* ReCiPe 2016 v1.1: A harmonized life cycle impact assessment method at midpoint and endpoint level. Report I: Characterization. RIVM Report 2016-0104a. (2017).

7. Vieira, M. D. M., Ponsioen, T. C., Goedkoop, M. J. & Huijbregts, M. A. J. Surplus Cost Potential as a Life Cycle Impact Indicator for Metal Extraction. *Resources 2016* **5**, 2 (2016).

8. Vieira, M. D. M. & Huijbregts, M. A. J. Comparing mineral and fossil surplus costs of renewable and non-renewable electricity production. *International Journal of Life Cycle Assessment* **23**, 840–850 (2018).

9. Springmann, M. *et al.* The healthiness and sustainability of national and global food based dietary guidelines: modelling study. *BMJ* **370**, m2322 (2020).

10. Poore, J. & Nemecek, T. Reducing food's environmental impacts through producers and consumers. *Science (1979)* **360**, 987–992 (2018).

11. Crippa, M. *et al.* Food systems are responsible for a third of global anthropogenic GHG emissions. *Nat Food* **2**, 198–209 (2021).

12. U.S. Energy Information Administration. T5.a Residential average monthly bill by Census Division, and State. *Electric Sales, Revenue, and Average price* https://www.eia.gov/electricity/sales_revenue_price/ (2020).

13. Pew Research Centre. Average number of people per household. https://www.pewresearch.org/fact-tank/2019/10/01/the-number-of-people-in-the-average-u-s-household-is-going-up-for-the-first-time-in-over-160-years/#:~:text=In%202018%20there%20were%202.63,households%20is%20trailing%20population%20growth (2019).

14. U.K. Department for Business, E. & I. S. Average annual domestic electricity bills by home and non-home supplier (overall, 2018, home suppliers). *Annual domestic energy bills* https://www.gov.uk/government/statistical-data-sets/annual-domestic-energy-price-statistics (2020).

15. Office for National Statistics. Families and households in the UK: 2018. https://www.gov.uk/government/statistical-data-sets/annual-domestic-energy-price-statistics (2019).

16. Food and Agriculture Organization of the United Nations (FAO). Food Balance Sheets. http://www.fao.org/faostat/en/#data/FBS (2018).

17. U.S. Energy Information Administration. U.S. Natural Gas Consumption by End Use. Total Consumption: Residential, 2018. (2020).

18. U.K. Department for Business, E. & I. S. Average annual domestic gas bills by home and non-home supplier (cash terms, home suppliers, 2018). *Annual domestic energy price statistics* https://www.gov.uk/government/statistical-data-sets/annual-domestic-energy-price-statistics (2020).

19. United States Environmental Protection Agency. WaterSense: Statistics and Facts. https://www.epa.gov/watersense/statistics-and-facts.

20. Energy Saving Trust. *At Home with Water*. https://www.energysavingtrust.org.uk/sites/default/files/reports/AtHomewithWater%287%29.pdf (2013).

21. U.S. Bureau of Transportation Statistics. U.S. Passenger Miles. https://www.bts.gov/content/us-passenger-miles (2020).

22. U.K. Department for Transport. TSGB0101: Passenger Transport by mode from 1952. *Modal comparisons (TSGB01)* https://www.gov.uk/government/statistical-data-sets/tsgb01-modal-comparisons#passenger-transport (2020).

23. Graver, B., Zhang, K. & Rutherford, D. *CO2 emissions from commercial aviation, 2018*. https://theicct.org/publications/co2-emissions-commercial-aviation-2018 (2019).

24. Willett, W. *et al.* Food in the Anthropocene: the EAT–Lancet Commission on healthy diets from sustainable food systems. *The Lancet* **393**, 447–492 (2019).

25. Aune, D. *et al.* Fruit and vegetable intake and the risk of cardiovascular disease, total cancer and all-cause mortality—a systematic review and dose-response meta-analysis of prospective studies. *Int J Epidemiol* **46**, 1029–1056 (2017).

26. Afshin, A., Micha, R., Khatibzadeh, S. & Mozaffarian, D. Consumption of nuts and legumes and risk of incident ischemic heart disease, stroke, and diabetes: a systematic review and meta-analysis. *Am J Clin Nutr* **100**, 278–288 (2014).

27. Bechthold, A. *et al.* Food groups and risk of coronary heart disease, stroke and heart failure: A systematic review and dose-response meta-analysis of prospective studies. *Crit Rev Food Sci Nutr* **59**, 1071–1090 (2019).

28. Schwingshackl, L. *et al.* Food groups and risk of colorectal cancer. *Int J Cancer* **142**, 1748–1758 (2018).

29. Schwingshackl, L. *et al.* Food groups and risk of type 2 diabetes mellitus: a systematic review and meta-analysis of prospective studies. *Eur J Epidemiol* **32**, 363–375 (2017).

30. Micha, R. *et al.* Etiologic effects and optimal intakes of foods and nutrients for risk of cardiovascular diseases and diabetes: Systematic reviews and meta-analyses from the Nutrition and Chronic Diseases Expert Group (NutriCoDE). *PLoS One* **12**, e0175149 (2017).

31. Springmann, M., Mozaffarian, D., Rosenzweig, C. & Micha, R. *Chapter 2. What we eat matters: Health and environmental impacts of diets worldwide. 2021 Global Nutrition Report: The state of global nutrition*. https://globalnutritionreport.org/reports/2021-global-nutrition-report/health-and-environmental-impacts-of-diets-worldwide/ (2021).

32. Development Initiatives. *2021 Global Nutrition Report: The state of global nutrition. Global Nutrition Report* https://globalnutritionreport.org/reports/2021-global-nutrition-report/ (2021).

33. Global Health Data Exchange. Global Burden of Disease Study 2018 Data Resources. https://ghdx.healthdata.org/gbd-2019 (2019).

34. Kastner, T., Kastner, M. & Nonhebel, S. Tracing distant environmental impacts of agricultural products from a consumer perspective. *Ecological Economics* **70**, 1032–1040 (2011).

35. Springmann, M. *et al.* Health and nutritional aspects of sustainable diet strategies and their association with environmental impacts: a global modelling analysis with country-level detail. *Lancet Planet Health* **2**, e451–e461 (2018).

36. Stylianou, K. S., Fulgoni, V. L. & Jolliet, O. Small targeted dietary changes can yield substantial gains for human health and the environment. *Nat Food* **2**, 616–627 (2021).

37. Nelson, G. *et al.* Income growth and climate change effects on global nutrition security to mid-century. *Nat Sustain* **1**, 773–781 (2018).

38. Weidema, B. P. Using the budget constraint to monetarise impact assessment results. *Ecological Economics* **68**, 1591–1598 (2009).

39. Weidema, B. P. Comparing Three Life Cycle Impact Assessment Methods from an Endpoint Perspective. *J Ind Ecol* **19**, 20–26 (2015).
