# Supplementary material for: Low-carbon diets can reduce global ecological and health costs
Source: Nat Food. 2023 May 15;4(5):394–406. doi: 10.1038/s43016-023-00749-2 (PMC10208974; doi:10.1038/s43016-023-00749-2)
Supplement: Supplementary file 2 — Reporting Summary [file 43016_2023_749_MOESM2_ESM.pdf]

Corresponding author(s): Gonzalo Guillén-Gosálbez

Last updated by author(s): Apr 5, 2023

Reporting Summary

Nature Research wishes to improve the reproducibility of the work that we publish. This form provides structure for consistency and transparency in reporting. For further information on Nature Research policies, see our Editorial Policies and the Editorial Policy Checklist.

Statistics

For all statistical analyses, confirm that the following items are present in the figure legend, table legend, main text, or Methods section.

n/a	Confirmed	
☐	☒	The exact sample size (*n*) for each experimental group/condition, given as a discrete number and unit of measurement
☒	☐	A statement on whether measurements were taken from distinct samples or whether the same sample was measured repeatedly
☒	☐	The statistical test(s) used AND whether they are one- or two-sided<br>*Only common tests should be described solely by name; describe more complex techniques in the Methods section.*
☒	☐	A description of all covariates tested
☒	☐	A description of any assumptions or corrections, such as tests of normality and adjustment for multiple comparisons
☐	☒	A full description of the statistical parameters including central tendency (e.g. means) or other basic estimates (e.g. regression coefficient) AND variation (e.g. standard deviation) or associated estimates of uncertainty (e.g. confidence intervals)
☒	☐	For null hypothesis testing, the test statistic (e.g. *F*, *t*, *r*) with confidence intervals, effect sizes, degrees of freedom and *P* value noted<br>*Give P values as exact values whenever suitable.*
☒	☐	For Bayesian analysis, information on the choice of priors and Markov chain Monte Carlo settings
☒	☐	For hierarchical and complex designs, identification of the appropriate level for tests and full reporting of outcomes
☒	☐	Estimates of effect sizes (e.g. Cohen's *d*, Pearson's *r*), indicating how they were calculated

Our web collection on statistics for biologists contains articles on many of the points above.

Software and code

Policy information about availability of computer code

Data collection	Food item damage and impact intensities (impact per kilogram of food) were calculated using SimaPro software V9.1.0.8 and then exported to Microsoft Excel V16.71.
Data analysis	Microsoft Excel V16.71, SimaPro V9.1.0.8 and MATLAB R2021b were used for the analysis and visualization of data.

For manuscripts utilizing custom algorithms or software that are central to the research but not yet described in published literature, software must be made available to editors and reviewers. We strongly encourage code deposition in a community repository (e.g. GitHub). See the Nature Research guidelines for submitting code & software for further information.

Data

Policy information about availability of data

All manuscripts must include a data availability statement. This statement should provide the following information, where applicable:

- Accession codes, unique identifiers, or web links for publicly available datasets
- A list of figures that have associated raw data
- A description of any restrictions on data availability

Food supply quantities and externalities results for all dietary change scenarios modelled in this study are available at: https://github.com/eglucas/LowCarbonDiets_Externalities. Select input data are also available in Supplementary Tables 8 – 13. Food supply data used in this study are available from the FAO Food Balance Sheets (http://www.fao.org/faostat/en/#data/FBS) and FCE data are available from the US Department of Agriculture Economic Research Service (https://www.ers.usda.gov/topics/international-markets-us-trade/international-consumer-and-food-industry-trends/#data). Life cycle inventory data for the calculation of food item impacts can be accessed in the commercially available databases ecoinvent (https://ecoinvent.org/the-ecoinvent-database/), Agri-Footprint (https://blonksustainability.nl/tools/agri-footprint) and ESU World Food (https://esu-services.ch/data/fooddata/).

Field-specific reporting

Please select the one below that is the best fit for your research. If you are not sure, read the appropriate sections before making your selection.

☐ Life sciences ☐ Behavioural & social sciences ☒ Ecological, evolutionary & environmental sciences

For a reference copy of the document with all sections, see nature.com/documents/nr-reporting-summary-flat.pdf

Ecological, evolutionary & environmental sciences study design

All studies must disclose on these points even when the disclosure is negative.

Study description	This study estimated the costs of human health burden, ecosystem quality reduction and damage to resource availability (i.e., externalities) from the environmental impacts of diets by combining national food supply quantities and environmental impact assessments of food items (via the ReCiPe2016 life cycle impact assessment method). Additionally, this study estimated the effect of changes in food consumption on human health using comparative risk assessment. Externalities were estimated for dietary patterns reported for 2018 and for nine modeled dietary change scenarios.
Research sample	Damage and impact intensities (impact per kilogram of food) were calculated for 708 food items available in the Ecoinvent V3.5, Agri-footprint V4 and ESU World Food life cycle inventory databases. Food supplies of 101 countries were analyzed.
Sampling strategy	The selection of food items were based on the 90 food groups reported in the FAO Food Balance Sheets for national food supply quantities. The diets and food supplies of 101 countries (representing 91% of the 2018 global population) were analyzed because they were the countries for which food supply and expenditure data were available for in both the FAO Food Balance Sheets and USDA Economic Research Service.
Data collection	No primary data collection was undertaken in this study and only secondary datasets were used. Publicly available national food supply data and Final Consumption Expenditure data were downloaded from FAOSTAT Food Balance Sheets and US Department of Agriculture Economic Research Service, respectively. Food item damage and impact intensities were calculated in SimaPro software V9.1, using commercially available life cycle inventory data from Ecoinvent V3, Agri-footprint V4 and ESU World Food databases. Relative risk parameters for dietary risk factor-disease pairs, as well as theoretical minimum risk exposure levels for dietary risk factors, were taken from various literature sources. All data were downloaded and compiled by Elysia Lucas.
Timing and spatial scale	2018 national food supply and Final Consumption Expenditure on food and non-alcoholic beverages of households data were downloaded from the FAOSTAT and USDA Economic Research Service websites, respectively, in April 2021.
Data exclusions	Supply quantities of alcoholic beverages reported in FAO Food Balance Sheets were excluded as disaggregated data of national Final Consumption Expenditure on alcoholic beverages were not available from the USDA Economic Research Service (only aggregated data of expenditure on alcoholic beverages and tobacco are provided).
Reproducibility	All sources of input data, life cycle impact assessment method and damage monetarization factors for externalities are disclosed. National food supply quantities in modeled dietary change scenarios are shared (https://github.com/eglucas/LowCarbonDiets_Externalities).
Randomization	Randomization is not relevant to this study as no experiments or trials were undertaken.
Blinding	Blinding is not relevant to this study as only publicly or commercially available data were used and all analysis was computational.

Did the study involve field work? ☐ Yes ☒ No

Reporting for specific materials, systems and methods

We require information from authors about some types of materials, experimental systems and methods used in many studies. Here, indicate whether each material, system or method listed is relevant to your study. If you are not sure if a list item applies to your research, read the appropriate section before selecting a response.

Materials & experimental systems

n/a	Involved in the study	
☒	☐	Antibodies
☒	☐	Eukaryotic cell lines
☒	☐	Palaeontology and archaeology
☒	☐	Animals and other organisms
☒	☐	Human research participants
☒	☐	Clinical data
☒	☐	Dual use research of concern

Methods

n/a	Involved in the study	
☒	☐	ChIP-seq
☒	☐	Flow cytometry
☒	☐	MRI-based neuroimaging
